# Respiratory syncytial virus co-opts hypoxia-inducible factor-1α-mediated glycolysis to favor the production of infectious virus

**DOI:** 10.1128/mbio.02110-23

**Published:** 2023-10-05

**Authors:** Li-Feng Chen, Jun-Xing Cai, Jing-Jing Zhang, Yu-Jun Tang, Jia-Yi Chen, Si Xiong, Yao-Lan Li, Hong Zhang, Zhong Liu, Man-Mei Li

**Affiliations:** 1 Department of Dermatology, The First Affiliated Hospital, Jinan University, Guangzhou, Guangdong, China; 2 Guangdong Province Key Laboratory of Pharmacodynamic Constituents of TCM and New Drugs Research, College of Pharmacy, Jinan University, Guangzhou, Guangdong, China; 3 Guangzhou Jinan Biomedicine Research and Development Center, Guangdong Provincial Key Laboratory of Bioengineering Medicine, College of Life Science and Technology, Jinan University, Guangzhou, Guangdong, China; Griffith University--Gold Coast Campus, Gold Coast, Australia

**Keywords:** respiratory syncytial virus, HIF-1α, glycolysis, IR-PI3K-Akt signaling, mitochondria, ROS

## Abstract

Glycolysis, a series of oxidative reactions used to metabolize glucose and provide energy to host cells, is also required for respiratory syncytial virus (RSV) infection. However, the role of glycolysis during RSV infection and its underlying molecular mechanisms remain to be further explored. In this study, we investigated the function of hypoxia-inducible factor (HIF)-1α-mediated glycolysis in HEp-2 cells and mouse models during RSV infection. The results showed that RSV infection activated the insulin receptor (IR)-PI3K-Akt axis, upregulated the translation and activity of HIF-1α, increased the expression of glucose transporters (Glut1, Glut3, and Glut4), hexokinase (HK) 1 and 2, and platelet-type phosphofructokinase (PFKP), and promoted glucose uptake and glycolysis. In addition, mitochondrial damage induced by RSV resulted in the generation of large amounts of reactive oxygen species (ROS) in infected cells, which contributed to the stabilization and activation of HIF-1α. An energy map of the glycolytic ATP production rate (Glyco-ATP) versus the mitochondrial ATP production rate (mito-ATP) confirmed a switch from oxidative phosphorylation (OXPHOS) to glycolysis. Inhibition of IR-PI3K-Akt signaling, ROS, or HIF-1α effectively reversed the RSV-induced increase in glycolysis by blocking HIF-1α activation. Importantly, HIF-1α-mediated glycolysis provided energy for the production of progeny RSV virions. The production of infectious virions was nearly abolished after knocking down HIF-1α. PX-478, an orally active HIF-1α inhibitor, effectively inhibited RSV infection *in vivo*. Collectively, these results indicate the role of HIF-1α-mediated glycolysis in RSV infection and highlight HIF-1α as a potential target for anti-RSV drug development.

**IMPORTANCE:**

Respiratory syncytial virus (RSV) is the leading etiological agent of lower respiratory tract illness. However, efficacious vaccines or antiviral drugs for treating RSV infections are currently not available. Indeed, RSV depends on host cells to provide energy needed to produce progeny virions. Glycolysis is a series of oxidative reactions used to metabolize glucose and provide energy to host cells. Therefore, glycolysis may be helpful for RSV infection. In this study, we show that RSV increases glycolysis by inducing the stabilization, transcription, translation, and activation of hypoxia-inducible factor (HIF)-1α in infected cells, which is important for the production of progeny RSV virions. This study contributes to understanding the molecular mechanism by which HIF-1α-mediated glycolysis controls RSV infection and reveals an effective target for the development of highly efficient anti-RSV drugs.

## INTRODUCTION

Human respiratory syncytial virus (RSV), a negative-sense single-stranded RNA virus of the *Paramyxoviridae* family, is the leading etiological agent of lower respiratory tract illness among young children, the elderly, and immunosuppressed adults, with 33 million infections and 66,000 deaths worldwide per year (1 – 4). However, efficacious vaccines for treating RSV infections are currently not available. Palivizumab and ribavirin are the only prophylactic and therapeutic agents approved for RSV infection, respectively, both of which are recommended for use only in high-risk patients, albeit with accompanying concerns of potential toxic side effects or cost-effectiveness (5, 6). Similar to all viruses, RSV is a non-cellular biological entity that depends on host cells to provide the molecular precursors, energy, and specialized components needed to produce progeny virions (7). Cellular metabolism caters to the metabolic and energy needs of cells (8). In the process of infecting cells, RSV has evolved multiple mechanisms to hijack the metabolic resources of the host for replication (9). Therefore, understanding how RSV manipulates cellular metabolism is critical for the development of effective vaccines or anti-RSV drugs.

Glucose metabolism is the main source of energy for host cells. Glycolysis and oxidative phosphorylation (OXPHOS) are a series of oxidative reactions used to metabolize glucose and provide energy to host cells. Once OXPHOS is impaired, glycolysis becomes the main source of ATP production. Hypoxia-inducible factor-1α (HIF-1α) is an oxygen-sensing transcription factor that determines whether glucose is consumed via OXPHOS or glycolysis (10). In host cells, the Warburg effect (switch from OXPHOS to glycolysis) is upregulated along with HIF-1α activation (11, 12). Under normal conditions, HIF-1α is rapidly hydroxylated by prolyl hydroxylases (PHDs) and degraded via ubiquitin-mediated proteasomal degradation. Hydroxylation is inhibited by hypoxia, allowing HIF-1α stabilization and its subsequent nuclear translocation. In the nucleus, HIF-1α dimerizes with HIF-1β and recognizes hypoxia-response elements (HREs) to drive the expression of target genes such as glucose transporter *GLUT1*, hexokinase 2 (*HK2*), and lactate dehydrogenase (*LDH*), eventually leading to increased glucose uptake and glycolysis (13 – 17). Currently, HIF-1α activity is regulated by reactive oxygen species (ROS) or the phosphoinositide-3 kinase (PI3K)/Akt signaling pathway (18 – 20). Increasing evidence suggests that in many cases, viral infection of host cells depends on HIF-1α-mediated glycolysis. HIF-1α regulates white spot syndrome virus (WSSV)-induced glycolysis by promoting the expression of glycolytic genes in white shrimp *Litopenaeus vannamei* (21). The use of mTORC1/eIF4E/HIF-1α pathways by avian reovirus (ARV) to promote virus replication is achieved by upregulating glycolysis and the TCA cycle (22). Moreover, it was reported that SARS-CoV-2 induces high glycolysis of monocytes through a HIF-1α/glycolysis-dependent axis, thereby promoting the production of progeny viruses (23). The relationship between RSV infection and HIF-1α-mediated glycolysis has also been preliminarily studied (24). However, further research is required to elucidate the underlying molecular mechanism by which HIF-1α-mediated glycolysis regulates RSV replication.

In this study, we investigated the function of HIF-1α-mediated glycolysis during RSV infection. The findings indicated that RSV infection increased HIF-1α-mediated glycolysis via activation of the insulin receptor (IR)-PI3K-Akt axis or upregulation of ROS levels. Inhibition of IR-PI3K-Akt signaling, ROS, or HIF-1α effectively reversed the RSV-induced increase in glycolysis by blocking HIF-1α activation. Furthermore, HIF-1α-mediated glycolysis provided energy for the production of progeny RSV virions. The production of RSV virions was nearly abolished after knocking down HIF-1α. PX-478 effectively inhibited RSV infection *in vivo*. These results indicate the role of HIF-1α-mediated glycolysis in RSV infection and highlight HIF-1α as a potential target for anti-RSV drug development.

## RESULTS

### RSV infection elevates glucose uptake and glycolysis in infected cells

**Fig 1:**
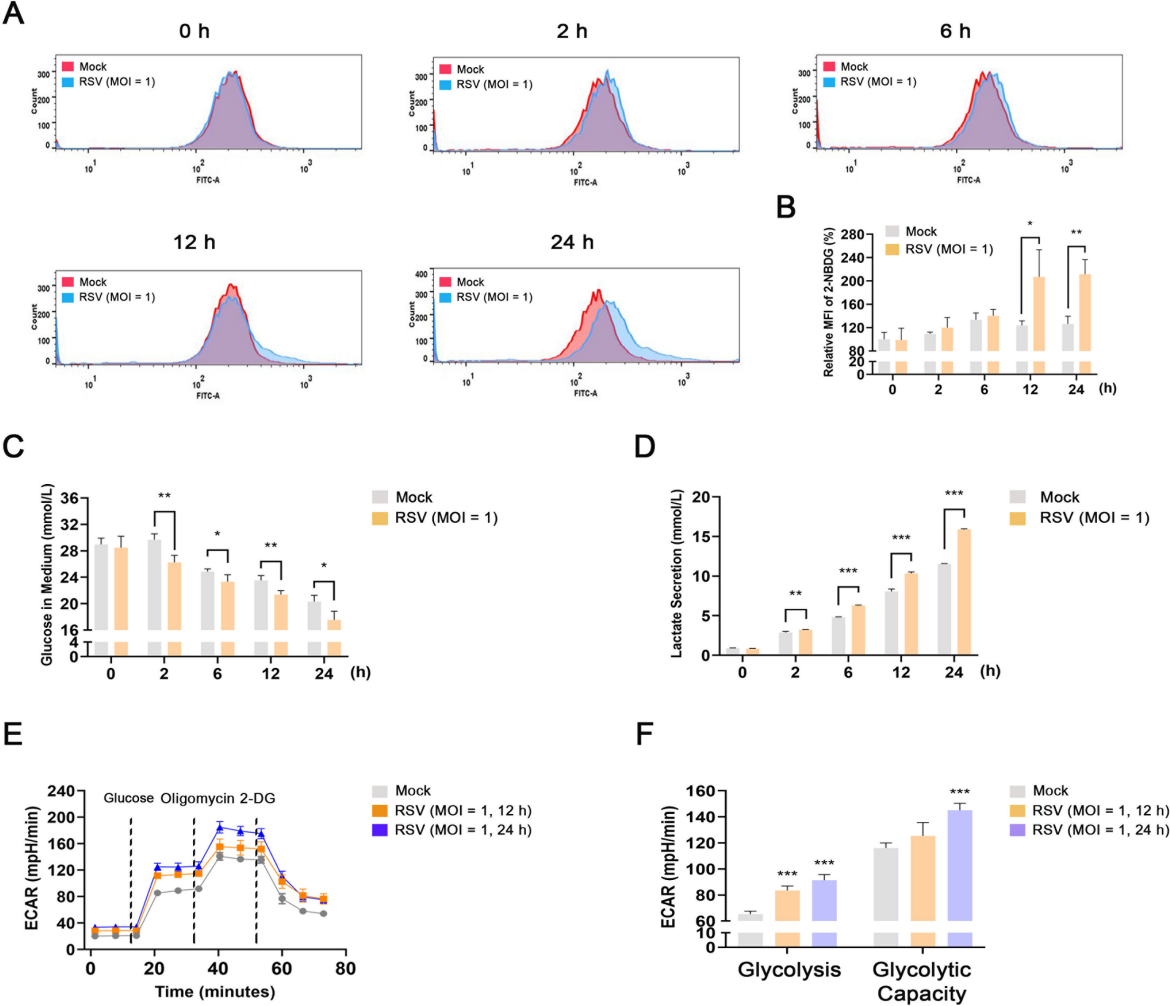
RSV infection elevates glucose uptake and glycolysis in infected cells. HEp-2 cells were mock-infected or infected with RSV (MOI = 1) for 0, 2, 6, 12, or 24 h. (**A and B**) Glucose uptake was measured using the 2-NBDG glucose uptake assay kit. (**C and D**) Glucose and lactic acid concentrations in the culture supernatant were detected using the glucose oxidation assay kit and the lactic acid assay kit, respectively. (**E and F**) Glycolytic activity was determined using the Seahorse XF glycolysis stress test kit. Data are shown as mean ± SD of at least three biological replicates, statistical analysis using Student’s *t* test (**B–D**) or one-way ANOVA (**F**). (*P < 0.05, **P < 0.01, ***P < 0.001 compared to the blank control group).

To investigate whether RSV infection increases glycolysis, HEp-2 cells were infected with RSV to determine glucose uptake using a fluorescently tagged glucose tracer, 2-NBDG, at different time intervals following RSV infection. The results showed that RSV significantly elevated glucose uptake in HEp-2 cells 12 and 24 h after infection (Fig. 1A and B). Next, we detected glucose consumption in RSV-infected cells. As shown in Fig. 1C, the glucose concentration in the culture medium of RSV-infected HEp-2 cells decreased more rapidly than that of mock-infected cells. To further validate the glucose uptake for increased glycolysis, the content of the glycolytic metabolite, lactic acid, was examined. As expected, RSV-infected cells secreted more lactic acid into the culture medium than mock-infected cells in a time-dependent manner (Fig. 1D). Additionally, the extracellular acidification rate (ECAR), a marker of glycolysis, was measured using the Seahorse XF glycolysis stress test kit. Basal levels of glycolysis were initially detected, followed by treatment with glucose (14.27 min) to examine the glycolytic flux. Oligomycin (33.84 min) was then administered to inhibit mitochondrial ATP production and utilize glycolysis for energy production. Finally, a glucose analog, 2-DG (53.41 min), was added to inhibit glycolysis. As shown in Fig. 1E and Fig. S1A, ECARs increased significantly in RSV-infected HEp-2 or 16HBE cells following the addition of glucose compared to that in mock-infected cells. The ECAR levels were further elevated in RSV-infected cells following oligomycin administration. Both RSV-infected and mock-infected cells showed significantly reduced ECAR levels after 2-DG treatment. Correspondingly, RSV-infected cells showed a robust increase in glycolysis and glycolytic capacity compared with mock-infected cells (Fig. 1F; Fig. S1B). The virus yield assay showed that the increase of progeny virions mainly occurred 12–48 h after RSV infection, which was consistent with the time point of upregulation of glycolysis (Fig. S2). Collectively, these data confirm that RSV infection increases glucose uptake and glycolysis in infected cells.

### RSV infection increases the expression of Glut1, Glut3, Glut4, HK1, HK2, and PFKP in infected cells

**Fig 2:**
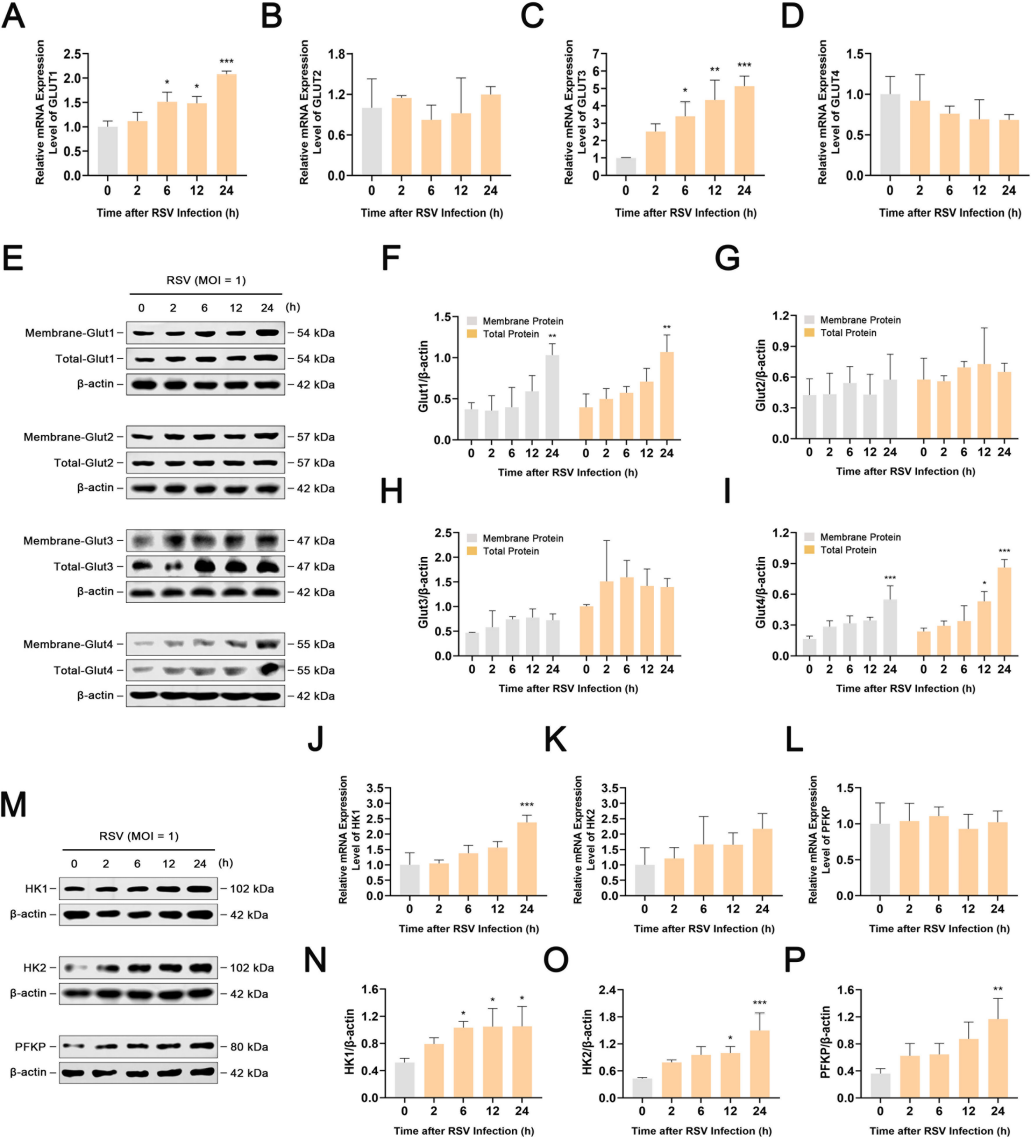
RSV infection increases the expression of Glut1, Glut3, Glut4, HK1, HK2, and PFKP in infected cells. HEp-2 cells were infected with RSV (MOI = 1) for 0, 2, 6, 12, or 24 h. (**A–D, J–L**) The mRNA levels of *GLUT1*, *GLUT2*, *GLUT3*, *GLUT4*, *HK1*, *HK2*, and *PFKP* were detected using RT-PCR. (**E–I, M–P**) Western blot analysis of Glut1, Glut2, Glut3, Glut4, HK1, HK2, and PFKP in the membrane or total protein. Data are shown as mean ± SD of three biological replicates, statistical analysis using one-way ANOVA (*P < 0.05, **P < 0.01, and ***P < 0.001 compared to the 0 h group).

Gluts are the main carriers mediating transmembrane transport of glucose, fructose, and xylose in mammalian cells. Among them, class I Gluts, including Glut1, Glut2, Glut3, and Glut4, are mainly responsible for transporting glucose (25 – 27). We next investigated whether the increase in glucose uptake in RSV-infected HEp-2 cells is due to alterations in Glut1–4 levels. The results showed that RSV infection progressively elevated the mRNA levels of *GLUT1* and *GLUT3* in infected cells in a time-dependent manner. Consistent with these data, the protein levels of Glut1 and Glut3 were increased in RSV-infected cells. Moreover, we also observed an increase in the protein level of Glut4 24-h post-infection, while its mRNA level did not change significantly. In contrast, RSV infection of HEp-2 cells did not affect Glut2 (Fig. 2A through I). As Gluts must migrate from the cytoplasm to the cell membrane for glucose uptake (28, 29), we further determined whether RSV promotes the migration of Glut1, Glut3, and Glut4 to the cell membrane of infected cells using a membrane and cytosol protein extraction kit. As shown in Fig. 2E, F, H and I, western blot analysis confirmed the enrichment of Glut1, Glut3, and Glut4 in the membrane fraction of RSV-infected cells, indicating significant translocation of Glut1, Glut3, and Glut4 to the cell membrane following RSV infection. The promotion of RSV on the expression of Glut1, Glut3, and Glut4 and their translocation to the cell membrane were also verified using immunofluorescence assays (Fig. S3). Collectively, these results suggest that RSV infection promotes glucose uptake by increasing the expression of Glut1, Glut3, and Glut4 and their translocation to the cell membrane.

Glycolysis is tightly controlled by a series of key glycolytic enzymes, including HK, phosphofructokinase (PFK), and pyruvate kinase (PK) (30 – 32). Considering that glycolysis increased in HEp-2 cells during RSV infection, we inferred that infection possibly elevates the expression levels of glycolytic enzymes. We then determined the mRNA and protein levels of HK1, HK2, PFKP, PKM, and LDHA using reverse transcription-PCR (RT-PCR) and western blot analysis, respectively. As shown in Fig. 2J through P, HEp-2 cells infected with RSV expressed higher mRNA levels of *HK1* and *HK2*. Accordingly, the results of western blotting showed that RSV infection in HEp-2 cells induced higher expression of HK1 and HK2. Moreover, we also observed an increase in the protein level of PFKP 24-h post-infection, while its mRNA level did not change significantly. In contrast, significant differences in PKM1/2, PKM2, and LDHA levels were not observed at different time intervals after RSV infection (Fig. S4). These data suggest that RSV infection promotes glycolysis by increasing the expression of HK1, HK2, and PFKP.

### RSV infection enhances the stability, expression, and transcriptional activity of HIF-1α in infected cells

**Fig 3:**
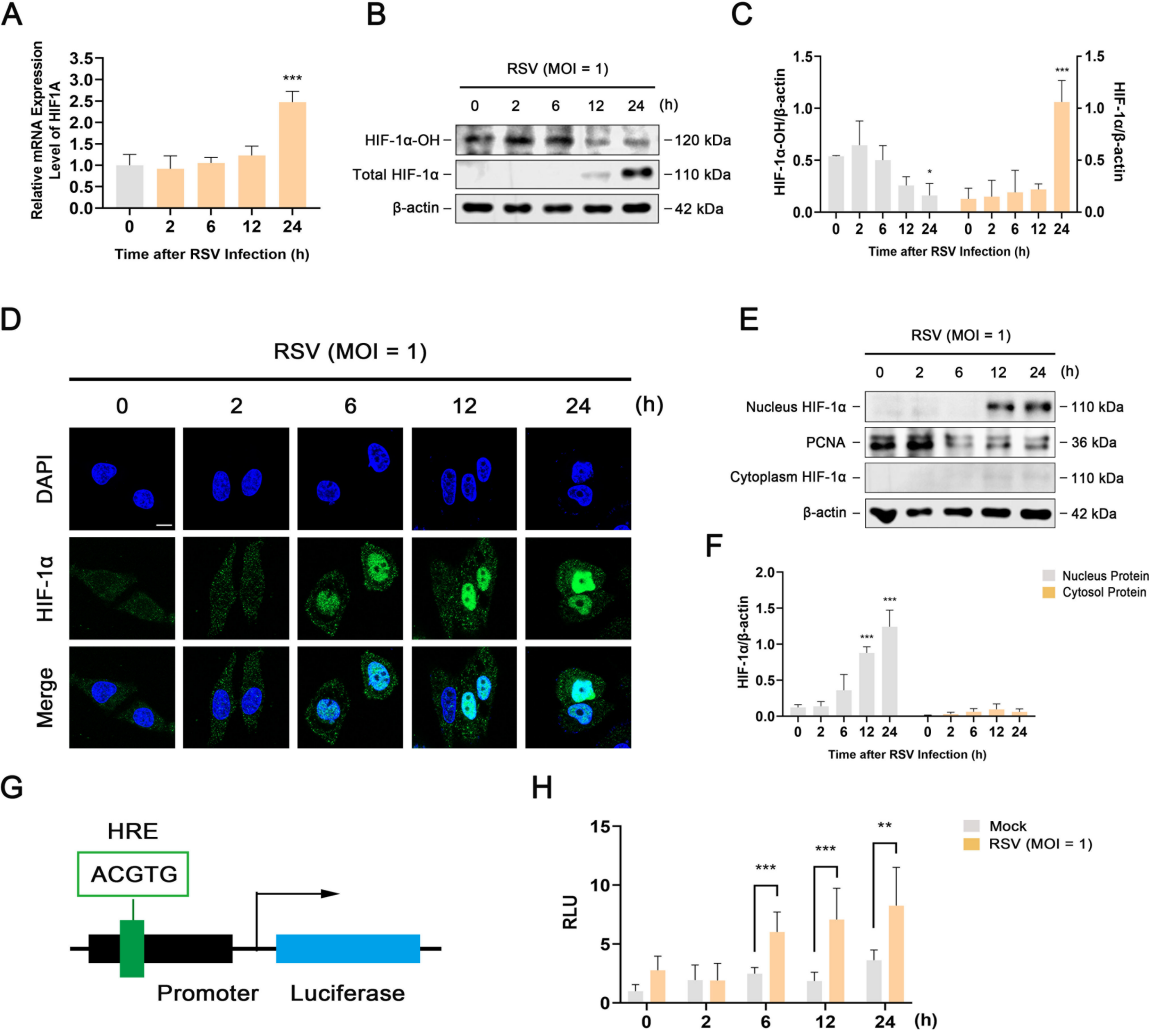
RSV infection enhances the stability, expression, and transcriptional activity of HIF-1α in infected cells. HEp-2 cells were mock-infected or infected with RSV (MOI = 1) for 0, 2, 6, 12, or 24 h. (**A**) The mRNA level of *HIF1A* was detected using RT-PCR. (**B and C**) Western blot analysis of hydroxylated HIF-1α (HIF-1α-OH) and total HIF-1α. (**D**) Immunofluorescence analysis of HIF-1α localization. Scale bar: 10 µm. (**E and F**) Western blot analysis of nuclear and cytosolic fractions of HIF-1α. PCNA (proliferating cell nuclear antigen) was used as the internal reference of nuclear protein. (**G and H**) HIF-1α activity was detected using the dual-luciferase reporter assay system. Data are shown as mean ± SD of at least three biological replicates, statistical analysis using Student’s *t* test (**H**) or one-way ANOVA (**A, C, and F**) (*P < 0.05, **P < 0.01, ***P < 0.001 compared to the 0 h group or the blank control group).

As HIF-1α acts as a core factor regulating glycolysis and controls the expression of Gluts and glycolytic enzymes (13 – 17), we reasoned that RSV may increase glycolysis by regulating HIF-1α in infected cells. To test this hypothesis, we first determined whether RSV infection upregulates the stability and expression of HIF-1α. As shown in Fig. 3A, RSV infection progressively elevated the mRNA level of *HIF1A* in infected cells in a time-dependent manner. Accordingly, RSV-infected HEp-2 cells accumulated more HIF-1α, while the level of hydroxylated HIF1α was significantly reduced, indicating that RSV infection contributes to the stabilization and expression of HIF-1α (Fig. 3B and C). Next, confocal microscopy images of HIF-1α staining revealed that HIF-1α was mainly dispersed throughout the cytoplasm 0–2 h after RSV infection. Most HIF-1α translocated from the cytoplasm to the nucleus in RSV-infected cells 12 and 24 h after infection (Fig. 3D). To confirm these results, we further detected the localization and trafficking of HIF-1α in RSV-infected HEp-2 cells using a nuclear protein extraction kit. As shown in Fig. 3E and F, western blot analysis confirmed the enrichment of HIF-1α in the nuclear fraction rather than in the cytoplasm of RSV-infected cells, indicating significant translocation of HIF-1α to the nucleus. Additionally, we constructed a luciferase reporter plasmid with a promoter containing the HRE sequence to determine the transcriptional activity of HIF-1α in RSV-infected cells. The results showed that RSV induced higher firefly luciferase activity in infected cells than in control cells 6, 12, and 24 h after infection (Fig. 3G and H). Taken together, these results indicate that RSV infection enhances the stability, expression, and transcriptional activity of HIF-1α.

### HIF-1α inhibition reduces glucose uptake and glycolysis during RSV infection

**Fig 4:**
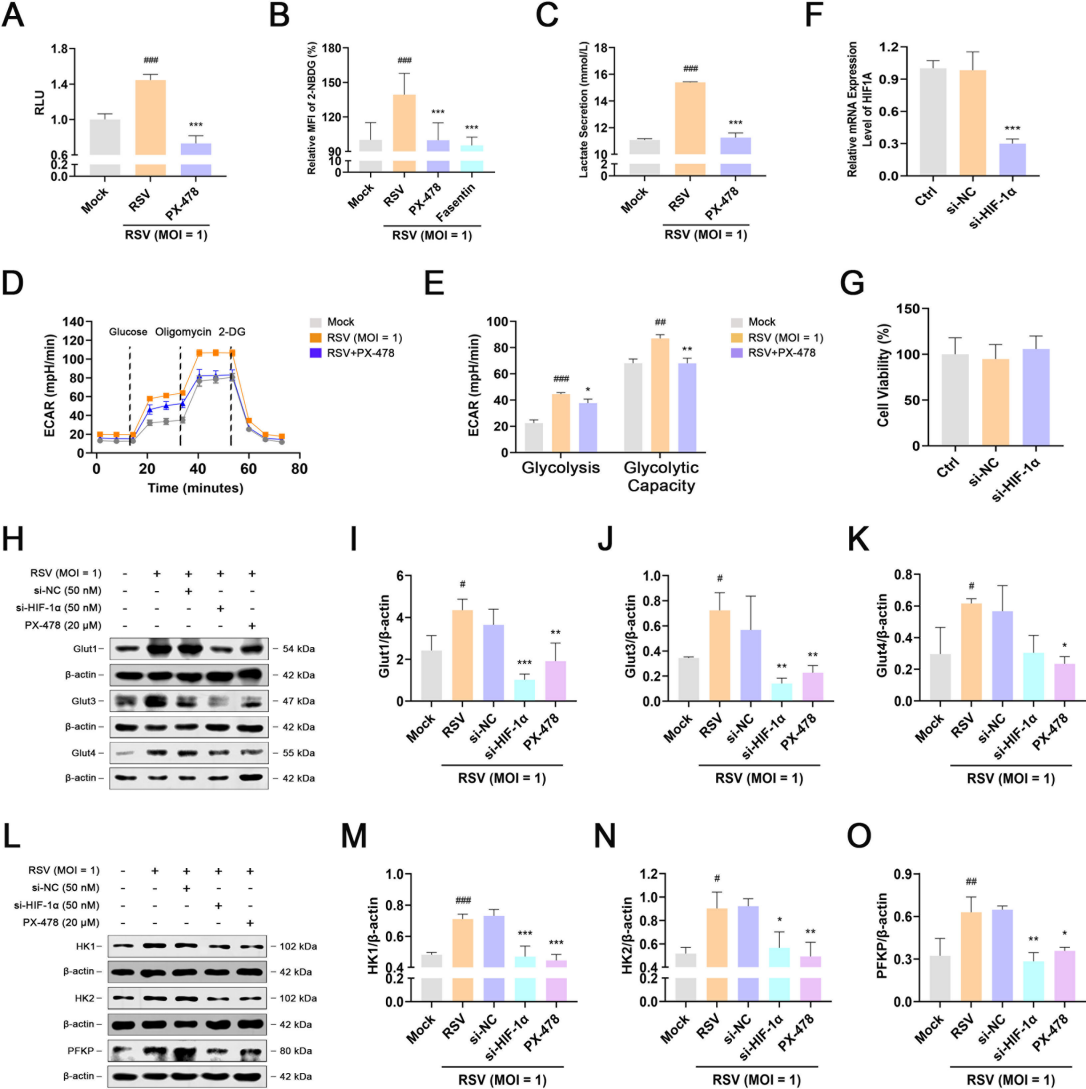
HIF-1α inhibition reduces glucose uptake and glycolysis during RSV infection. HEp-2 cells were mock-infected or infected with RSV (MOI = 1) in the presence or absence of PX-478 (20 µM), si-HIF-1α (50 nM), si-NC (negative control nucleotide, 50 nM), or fasentin (40 µM) for 24 h. (**A**) Effect of PX-478 on HIF-1α activity during RSV infection was detected using the dual-luciferase reporter assay system. (**B**) Effect of PX-478 or fasentin on glucose uptake during RSV infection was measured using the 2-NBDG glucose uptake assay kit. (**C**) Effect of PX-478 on lactic acid content in the culture supernatant during RSV infection was detected using the lactic acid assay kit. (**D and E**) Effect of PX-478 on glycolytic activity during RSV infection was determined using the Seahorse XF glycolysis stress test kit. (**F**) Effect of si-HIF-1α on the mRNA level of *HIF1A* in HEp-2 cells was detected using RT-PCR. (**G**) Cytotoxicity of si-HIF-1α against HEp-2 cells was measured using CCK-8 assay. (**H–O**) Effect of si-HIF-1α or PX-478 on the expression of Glut1, Glut3, Glut4, HK1, HK2, and PFKP during RSV infection was determined using western blot assay. Data are shown as mean ± SD of at least three biological replicates, statistical analysis using one-way ANOVA (^#^ *P* < 0.05, ^##^ *P* < 0.01, and ^###^ *P* < 0.001 compared to the blank control group; *P < 0.05, **P < 0.01, and ***P < 0.001 compared to the viral control group).

Next, we used an active HIF-1α inhibitor, PX478, combined with a siRNA specifically targeting HIF-1α to confirm the effect of HIF-1α on glycolysis during RSV infection. As shown in Fig. 4A; Fig. S5, treatment with PX-478 effectively reversed the expression and activation of HIF-1α induced by RSV infection. 2-NBDG uptake in infected cells increased significantly after RSV infection. PX-478 or fasentin (a specific inhibitor of Gluts) impaired RSV-induced glucose uptake (Fig. 4B). Compared to mock-treated cells, the rate of increase in ECAR levels was dramatically reduced in PX-478-treated cells following the administration of glucose or oligomycin, demonstrating the blocking effect of HIF-1α inhibition on glycolytic flux and the transition from OXPHOS to glycolysis during RSV infection. PX-478 treatment also decreased glycolysis and glycolytic capacity in RSV-infected cells, with a corresponding decrease in lactic acid level in the culture medium (Fig. 4C through E).

Next, we investigated whether inhibition of HIF-1α reduces RSV-induced increase in glycolysis by blocking the expression of Gluts and glycolytic enzymes. As shown in Fig. 4F and G, the HIF-1α-specific siRNA inhibited 70% of *HIF1A* mRNA expression at the concentration of 50 nM, as detected using RT-PCR. Nearly 100% of HEp-2 cells remained viable at the corresponding concentration, suggesting that si-HIF-1α does not show any obvious cytotoxicity. si-HIF-1α also reduced RSV-induced increase in HIF-1α protein levels (Fig. S5). Consistent with the data shown in Fig. 2, RSV-infected cells showed markedly higher levels of Glut1, Glut3, Glut4, HK1, HK2, and PFKP than mock-infected cells 24 h after infection. Knockdown of HIF-1α with si-HIF-1α or treatment with PX-478 resulted in almost complete inhibition of RSV-induced upregulation of Glut1, Glut3, Glut4, HK1, HK2, and PFKP in RSV replicon cells (Fig. 4H through O). In summary, we conclude that HIF-1α inhibition reduces glycolysis in RSV-infected cells.

### IR/PI3K/Akt signaling controls HIF-1α-mediated glycolysis by regulating HIF-1α translation during RSV infection

**Fig 5:**
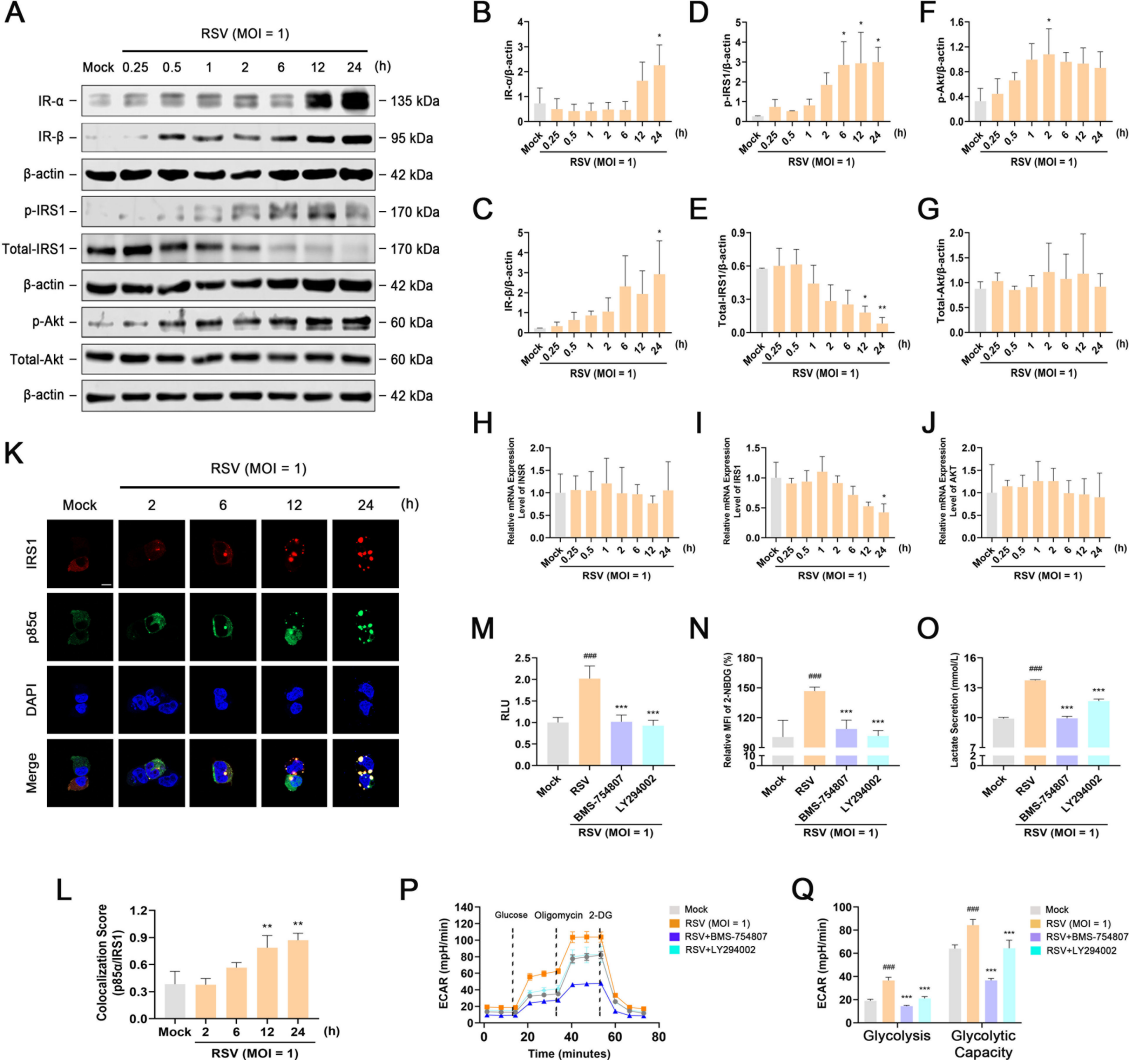
HIF-1α-mediated glycolysis is controlled by IR/PI3K/Akt signaling during RSV infection. HEp-2 or HEK-293T cells were mock-infected or infected with RSV (MOI = 1) in the presence or absence of BMS-754807 (25 µM) or LY294002 (10 µM) for the indicated durations. (**A–G**) Western blot analysis of IR-α, IR-β, p-IRS1, IRS1, p-Akt, and Akt in HEp-2 cells for 0.25, 0.5. 1, 2, 6, 12, or 24 h after RSV infection. (**H–J**) The mRNA levels of *INSR*, *IRS1*, and *AKT* in HEp-2 cells for 0.25, 0.5. 1, 2, 6, 12, or 24 h after RSV infection were detected using RT-PCR. (**K and L**) Immunolocalization of EGFP-p85α and mCherry-IRS1 in HEK293T cells for 2, 6, 12, or 24 h after RSV infection. Scale bar: 10 µm. (**M**) Effect of BMS-754807 or LY294002 on HIF-1α activity in HEp-2 cells 24 h after RSV infection was detected using the dual-luciferase reporter assay system. (**N**) Effect of BMS-754807 or LY294002 on glucose uptake in HEp-2 cells 24 h after RSV infection was measured using the 2-NBDG glucose uptake assay kit. (**O**) Effect of BMS-754807 or LY294002 on lactic acid content in the culture supernatant 24 hr after RSV infection was detected using the lactic acid assay kit. (**P and Q**) Effect of BMS-754807 or LY294002 on glycolytic activity in HEp-2 cells 24 h after RSV infection was determined using the Seahorse XF glycolysis stress test kit. Data are shown as mean ± SD of at least three biological replicates, statistical analysis using one-way ANOVA (^###^ *P* < 0.001 compared to the blank control group; *P < 0.05, **P < 0.01, and ***P < 0.001 compared to the blank control group or the viral control group).

PI3K/Akt signaling is closely related to cellular glycolysis levels (33). The PI3K/Akt axis is not only the main downstream pathway for IR to perform metabolic functions (34, 35), but also regulates the translation and activation of HIF-1α (19, 20, 36). Therefore, we inferred that IR/PI3K/Akt signaling participates in the regulation of glycolysis through HIF-1α during RSV infection. First, to evaluate whether RSV infection activates IR/PI3K/Akt signaling, we examined the mRNA and protein levels of IR, IRS1, and Akt in mock-infected and RSV-infected HEp-2 cells using RT-PCR and western blotting, respectively. As shown in Fig. 5A through G, RSV infection induced obvious increase in the levels of IR-α and IR-β in a time-dependent manner compared to that in mock-infected cells. Cellular IRS1 was significantly activated in RSV-infected cells to a level higher than that in mock-infected cells in a time-dependent manner. However, the total IRS1 level showed a decreasing trend (0.5–24 h) during RSV infection. Akt acts as the most important downstream effector of PI3K and is phosphorylated once PI3K signaling is activated. Therefore, Akt phosphorylation is considered a reliable indicator of PI3K signaling activation (37 – 39). RSV infection upregulated the phosphorylation level of Akt in a time-dependent manner, but did not alter the total level of Akt compared to that in mock-infected cells. Next, we analyzed the mRNA levels of *IR*, *IRS1*, and *AKT* using RT-PCR. The results showed that *INSR* and *AKT* mRNA levels were similar in both RSV-infected and mock-infected cells. The change in the mRNA level of *IRS1* coincided with the trend observed in its protein level (Fig. 5H through J). Moreover, it has been shown that the interaction between IRS1 and PI3K regulatory subunit, p85α, is essential for the activation of IR/PI3K/Akt signaling (40). Herein, we used co-immunolocalization to verify the relationship between IRS1 and p85α, and detected enhanced interaction between IRS1 and p85α following RSV infection (Fig. 5K and L). Based on these data, we conclude that IR/PI3K/Akt signaling is significantly activated following RSV infection.

To further validate whether IR/PI3K/Akt signaling is required for the increase in HIF-1α-mediated glycolysis during RSV infection, we treated RSV-infected cells with BMS-754807 or LY294002, specific inhibitors of IR or PI3K, respectively, and measured the expression and activity of HIF-1α, glucose uptake, lactic acid production, and glycolysis levels. As shown in Fig. 5M through Q and Fig. S6A through C, treatment of RSV-infected HEp-2 cells with BMS-754807 or LY294002 remarkably reduced the protein expression and activity of HIF-1α without affecting its mRNA level. As determined using glucose uptake and lactic acid production assays, the glucose uptake and lactic acid production in infected cells increased significantly 24 h after RSV infection. BMS-754807 and LY294002 treatment significantly inhibited the enhancement of these events following RSV infection. The results of the Seahorse XF glycolysis stress test assay showed that inhibition of IR or PI3K in RSV-infected cells dramatically reduced ECAR levels, glycolysis, and glycolytic capacity. These data suggest that HIF-1α-mediated glycolysis is controlled by IR/PI3K/Akt signaling during RSV infection.

Previous studies have identified that PI3K/Akt signaling regulates the protein synthesis of HIF-1α through its downstream effector, mTOR (41). mTOR mediates its action via phosphorylation of the eukaryotic translation initiation factor 4E (eIF-4E) binding protein (4E-BP1) (42). To evaluate whether PI3K/Akt signaling increases HIF-1α translation by mTOR and 4E-BP1 during RSV infection, we first determined the phosphorylation levels of these proteins. As shown in Fig. S6D through G, RSV infection upregulated the phosphorylation levels of mTOR and 4E-BP1 in a time-dependent manner, but did not alter the total level of mTOR. Treatment of RSV-infected HEp-2 cells with rapamycin (RAPA, a specific mTOR inhibitor) significantly reduced the protein expression and activity of HIF-1α, which could not be abolished by MG-132 (an inhibitor of the 26S proteasome: to prevent HIF-1α from being degraded) (Fig. S6H through J). In summary, IR/PI3K/Akt signaling upregulates the level of HIF-1α by enhancing HIF-1α translation rather than its transcription and stability.

### Upregulation of ROS caused by damaged mitochondria contributes to the stabilization and activation of HIF-1α during RSV infection

**Fig 6:**
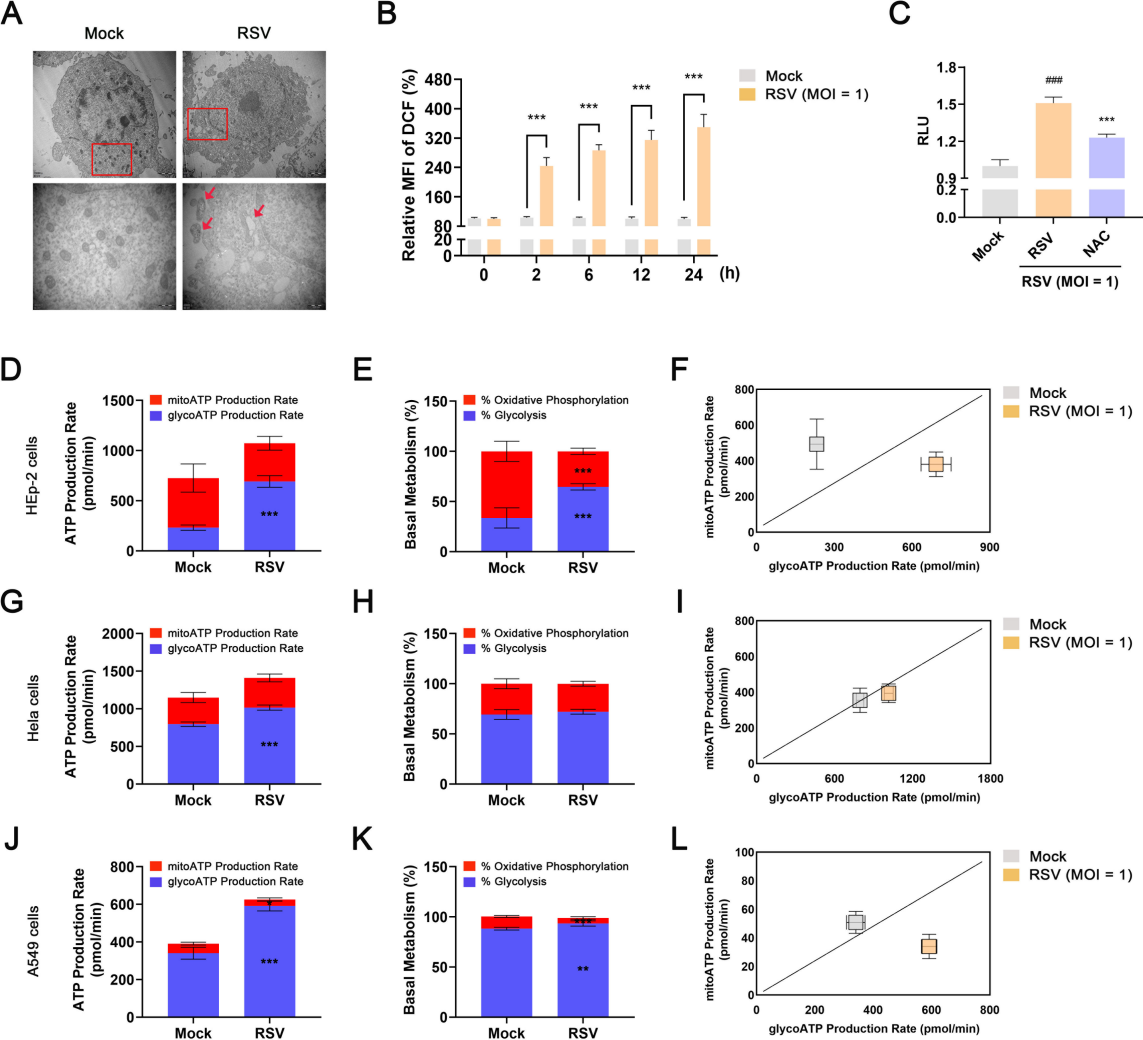
RSV infection impairs mitochondrial function, increases ROS production, and promotes a metabolic switch from OXPHOS to glycolysisin infected cells. HEp-2, Hela, or A549 cells were mock-infected or infected with RSV (MOI = 1) in the presence or absence of NAC (400 µM) for the indicated durations. (**A**) Morphology of mitochondria in HEp-2 cells 24 h after RSV infection was examined using TEM. Red arrows indicate the damaged mitochondria. Scale bar: 2 µm or 500 nm. (**B**) ROS levels in HEp-2 cells for 0, 2, 6, 12, or 24 h after RSV infection was detected using the ROS assay kit. (**C**) Effect of NAC on HIF-1α activity in HEp-2 cells 24 h after RSV infection was detected using the dual-luciferase reporter assay system. Analysis of metabolic flux [(D, G, J): total ATP production; (E, H, K): ATP derived from glycolysis or OXPHOS; (F, I, L): energetic map of mito-ATP versus Glyco-ATP] in HEp-2, Hela, or A549 cells 24 h after RSV infection was measured using the Seahorse XF real-time ATP rate test kit. Data are shown as mean ± SD of at least three biological replicates, statistical analysis using Student’s *t* test (**B, D–L**) or one-way ANOVA (**C**) (^###^ *P* < 0.001 compared to the blank control group; *P < 0.05, **P < 0.01, and ***P < 0.001 compared to the blank control group or the viral control group).

Previous studies have reported that ROS, which mainly originates from the mitochondria, regulates HIF-1α stability (18, 43). Once the mitochondria are impaired, the cells induce the production of large amounts of ROS. In this study, we used transmission electron microscopy (TEM) to find that RSV infection induced the formation of damaged mitochondria with collapsed cristae and matrix swelling. Moreover, the interaction between damaged mitochondria and autophagic vacuoles indicated the occurrence of mitophagy in infected cells (Fig. 6A). Accordingly, we also observed that the amount of ROS significantly increased in RSV-infected cells in a time-dependent manner (Fig. 6B). Treatment with acetylcysteine (NAC), a specific inhibitor of ROS, inhibited the expression and activation of HIF-1α by increasing the level of hydroxylated HIF1α (Fig. 6C ; Fig. S7). Thus, we conclude that RSV infection upregulates the stability and activity of HIF-1α by promoting ROS production.

### RSV infection promotes a metabolic switch from OXPHOS to glycolysis in infected cells

The majority of ATP in host cells is provided by the mitochondria. Upregulation of glycolysis can compensate for insufficient ATP synthesis once the mitochondrial function is impaired. To evaluate whether glycolysis-derived ATP is regulated by RSV, we selected three cell lines that are commonly used in RSV-related studies for real-time ATP synthesis: HEp-2, Hela, and A549. Results of the real-time ATP synthesis rate assay revealed that total ATP production in RSV-infected cells was higher than that in mock-infected cells (HEp-2, Hela, and A549). Moreover, we observed an increase in the proportion of ATP derived from glycolysis in infected HEp-2 cells with a decrease in the proportion of ATP derived from mitochondria. RSV infection also increased the Glyco-ATP production rate. An energy map of the glycolysis ATP production rate (Glyco-ATP) versus the mitochondrial ATP production rate (mito-ATP) showed a switch from OXPHOS to glycolysis. A similar phenomenon was observed in Hela and A549 cells, although the trend is not comparable to that in HEp-2 cells (Fig. 6D through L). Correspondingly, the promotion of RSV on HIF-1α expression in A549 cells was weaker than that in HEp-2 cells (Fig. S8). Overall, these results suggest that glycolysis, not mitochondrial function, is more essential for RSV infection.

### Inhibition of HIF-1α inhibits RSV infection *in vitro* and *in vivo*

**Fig 7:**
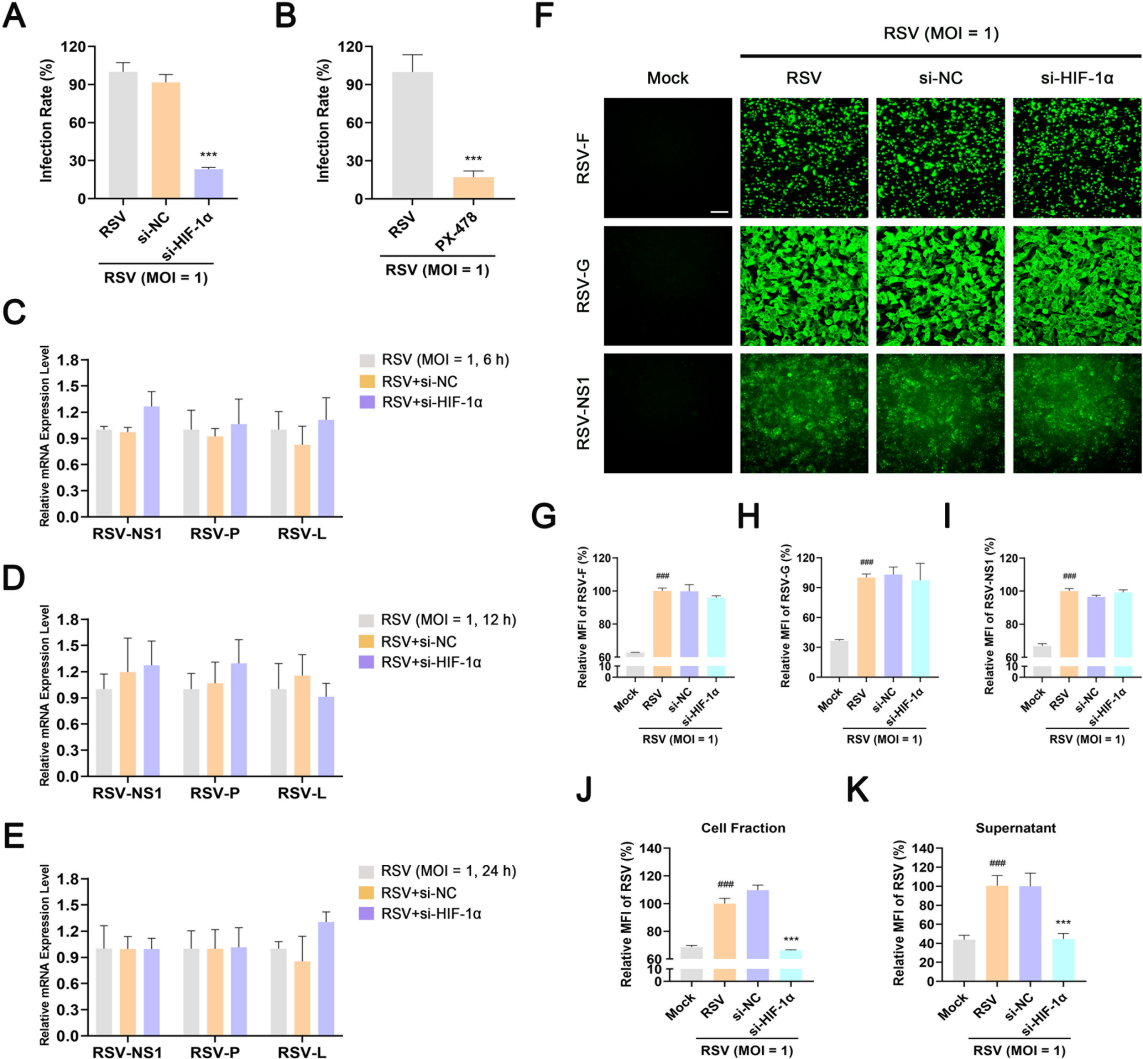
HIF-1α knockdown inhibits infectious virus production *in vitro*. HEp-2 cells were mock-infected or infected with RSV (MOI = 1) in the presence or absence of si-HIF-1α (50 nM), si-NC (50 nM), or PX-478 (20 µM) for the indicated durations. Effect of si-HIF-1α (**A**) or PX-478 (**B**) on viral titers in RSV-infected HEp-2 cells was measured using immunofluorescence assay. (**C–E**) Effect of si-HIF-1α on the mRNA levels of *RSV NS1*, *P*, and *L* for 6, 12, or 24 h after RSV infection was detected using RT-PCR. (**F–I**) Effect of si-HIF-1α on the protein levels of RSV NS1, G, and F 24 h after RSV infection was detected using immunofluorescence assay. Scale bar: 50 µm. (**J and K**) Effect of si-HIF-1α on the amount of progeny virus in the cell fraction or supernatant was detected using immunofluorescence assay. Data are shown as mean ± SD of at least three biological replicates, statistical analysis using Student’s *t* test (**B**) or one-way ANOVA (**A, C–E, and G–K**) (^###^ *P* < 0.001 compared to the blank control group; ***P < 0.001 compared to the viral control group).

To determine the effect of HIF-1α-mediated glycolysis in RSV infection, we used si-HIF-1α and PX-478 and performed a series of experiments to confirm whether HIF-1α controls RSV infection. As shown in Fig. 7A, after 48 h of incubation, the number of infectious viral particles decreased by 77% in si-HIF-1α-transfected cells, as observed in the immunofluorescence assay. In addition, PX-478 exerted anti-RSV activity equivalent to that of si-HIF-1α (Fig. 7B). The inhibitory effect of si-HIF-1α or PX-478 on RSV infection was also confirmed using plaque assays (Fig. S9A). These results indicate that HIF-1α plays a key role in RSV infection.

The life cycle of RSV involves the uptake of viral particles, release of the nucleocapsid and genome, and direct expression of the viral gene to produce viral proteins, followed by antigenome (positive-sense RNA) generation for the eventual synthesis of a new negative-sense genome and assembly and budding of progeny virions (44). RT-PCR, western blotting, and immunofluorescence assays were performed to investigate the stage at which si-HIF-1α exerts its inhibitory effect *in vitro*. As shown in Fig. 7C through E, the mRNA levels of *RSV NS1*, *P*, and *L* in si-HIF-1α-transfected cells did not change considerably for 6, 12, and 24 h after infection compared to that observed in the RSV control group, indicating that si-HIF-1α does not affect viral entry or viral gene transcription. Similarly, the levels of RSV G, F, and NS1 proteins did not differ significantly between the viral control and the si-HIF-1α-transfected group (Fig. 7F through I). To further determine whether si-HIF-1α works in the stage of progeny virion production, the cell fraction and supernatant were collected 48 h after RSV infection and the viral titer was measured using an immunofluorescence assay. The addition of si-HIF-1α significantly reduced viral titers in both the cell fraction and supernatant, suggesting that HIF-1α is indispensable for the production of progeny RSV virions (Fig. 7J and K). Moreover, the results of plaque assay showed that knockdown of HIF-1α significantly reduced the production of progeny RSV virions, whereas overexpression of HIF-1α had no effect on the production of progeny RSV virions (Fig. S9B and C).

**Fig 8:**
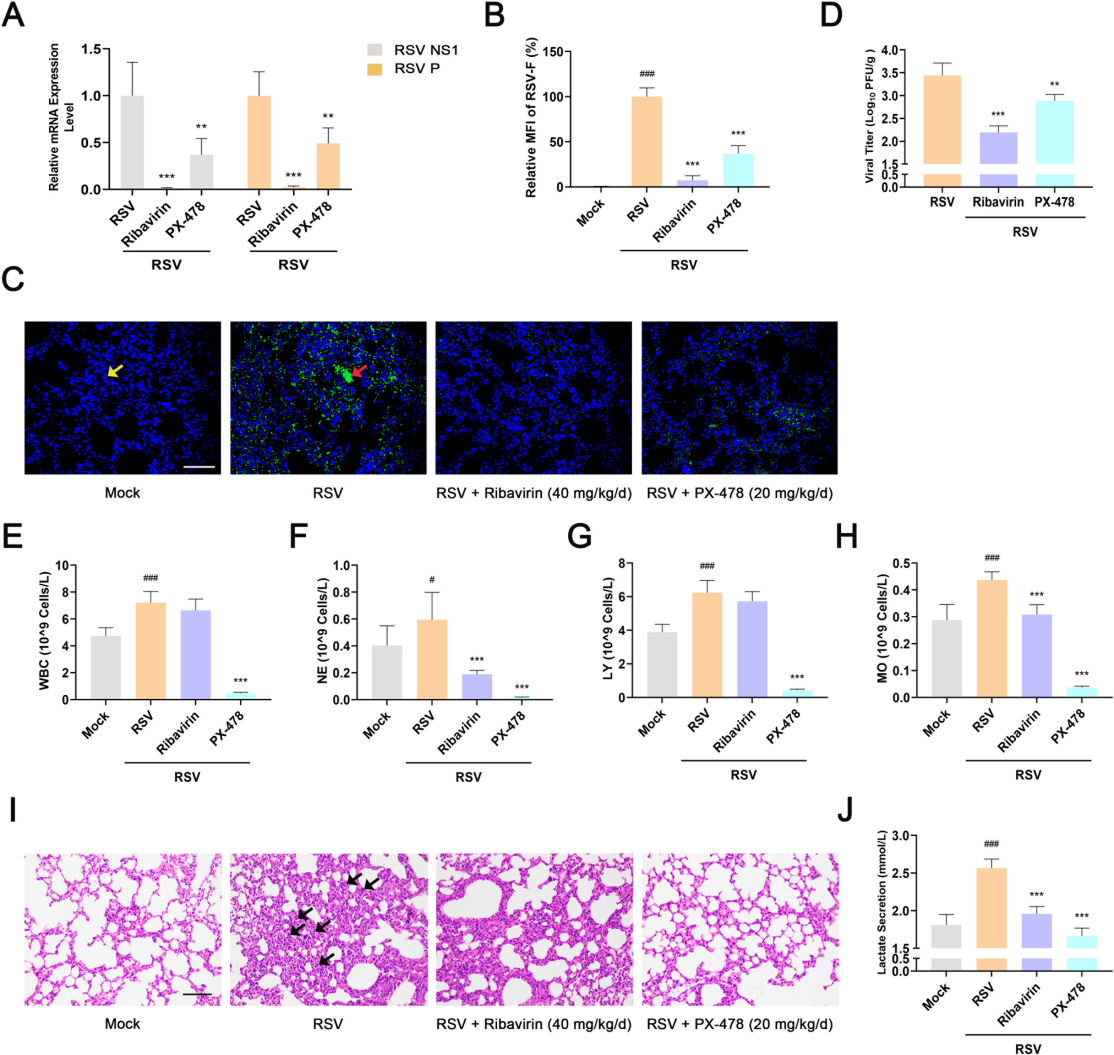
Inhibition of HIF-1α inhibits RSV infection *in vivo*. BALB/c mice were mock-infected or infected with RSV. PX-478 (20 mg·kg^−1^) was intragastrically administered before inoculation of RSV (−1 day) and then administered every 24 h (−1 to 3 days). On day 4 after RSV infection, the *RSV NS1* and *P* mRNA levels (**A**), RSV F protein level (**B and C**), and viral titers (**D**) in the lung tissues of each group were determined using RT-PCR, immunofluorescence assay, and plaque assay, respectively. Yellow arrow indicates DAPI. Red arrow indicates the RSV F protein. Scale bar: 100 µm. (**E–H**) The number of WBC, NE, LY, and MO in the blood of each group was measured using a five-part differential automated hematological analyzer. (**I**) Histopathological analysis of lung tissues in each group was performed using hematoxylin–eosin staining. Black arrow indicates the pulmonary infiltration of inflammatory cells. Scale bar: 100 µm. (**J**) Lactic acid in the lung tissues of each group was detected using the lactic acid assay kit. Data are shown as mean ± SD of at least three biological replicates, statistical analysis using one-way ANOVA (^#^ *P* < 0.05, ^###^ *P* < 0.001 compared to the blank control group; **P < 0.01 and ***P < 0.001 compared to the viral control group).

An RSV-infected mouse model was established to confirm the effect of HIF-1α on RSV infection *in vivo*. Briefly, mock-infected or RSV-infected mice were intragastrically treated with ribavirin (40 mg·kg^−1^), PX-478 (20 mg·kg^−1^), or normal saline once daily for 5 days. On day 4 post-infection, the mice in each group were sacrificed to measure the viral titers in the lung tissues. After treatment with PX-478 for 5 days, *RSV NS1* and *P* mRNA levels and RSV F protein levels in the lung tissues were lower than those in the virus group (Fig. 8A through C). In addition, the data from the plaque assay also suggested that PX-478 significantly reduced pulmonary viral titers, indicating that intragastric therapy with PX-478 can inhibit RSV multiplication in mice (Fig. 8D). Ribavirin treatment also reduced *RSV NS1* and *P* mRNA levels, RSV F protein levels, and viral titers in the lung tissues (Fig. 8A through D). To further determine the effects of PX-478 on viral pneumonia in mice, the lung tissues were subjected to histopathological analysis. Histopathological examination revealed that PX-478 effectively relieved RSV-induced hyperemia, pulmonary inflammatory infiltration, and alveolar damage in the lung tissues (Fig. 8I). Moreover, mice challenged with RSV showed aberrant elevation in the numbers of leukocytes (WBC), neutrophils (NE), lymphocytes (LY), and monocytes (MO) in the blood. PX-478 inhibited this phenomenon (Fig. 8E through H), and it showed better inhibition of inflammation caused by RSV than ribavirin. Importantly, PX-478 significantly reduced RSV-induced HIF-1α expression and lactic acid production in the lung tissues. Ribavirin also reduced the content of HIF-1α and lactic acid in the lung tissues, possibly due to reduced viral load (Fig. 8J; Fig. S10). Thus, we conclude that HIF-1α controls RSV infection by regulating glycolysis *in vivo*.

## DISCUSSION

As viruses depend on host cells to provide molecular precursors and energy for their successful infection, hijacking cellular metabolism is a common strategy used by viruses for their survival and persistence (45). As one of the main energy sources of host cells, glycolysis has been manipulated by many viruses to facilitate their own replication, such as herpes simplex virus 1 (HSV-1), dengue virus (DENV), and Zika virus (ZAKV) (46 – 48). Previous studies have also identified that RSV infection promotes glycolysis in primary human small alveolar epithelial cells or bone marrow-derived cells (24, 49). Consistent with these observations, we observed here that RSV infection increased glycolysis in HEp-2, 16HBE, and A549 cells. However, Codo et al. argued that glycolysis is not essential for RSV replication in monocytes (23). Indeed, various viruses participate in the regulation of glycolysis in host cells via different mechanisms. Several studies have reported that human HHV-6 promotes glycolysis in infected T cells by activating Akt-mTOR signaling (50). Influenza A Virus (H1N1) activates glycolysis by upregulating the expression of HK2, PKM2, and pyruvate dehydrogenase kinase 3 in infected cells (51). A recent study has shown that NDV induces SIRT3 degradation in damaged mitochondria via PINK1-PRKN-dependent mitophagy and shifts OXPHOS toward glycolysis, thereby promoting viral replication (52). Thus, defining the specific host cell glycolytic features required for different viruses may be helpful for the development of safe and effective antiviral therapies. So far, the glycolytic pathway has been shown to be associated with the autophagy protein ATG5 or HIF-1α during RSV infection, respectively (24, 49). Based on this, we further investigated glycolysis *in vitro* and *in vivo* during RSV infection in detail. The results showed that the increase in glycolysis in infected cells was due to RSV-induced mitochondrial damage, which led to a metabolic switch from OXPHOS to glycolysis. Moreover, RSV activated HIF-1α to increase the expression of related Gluts and glycolytic enzymes via the activation of IR/PI3K/Akt signaling or upregulation of ROS levels, thereby promoting glycolysis. Importantly, pharmacological inhibition of HIF-1α significantly inhibited RSV infection *in vitro* and *in vivo*, suggesting a potential anti-RSV target.

Glycolytic activity depends on the rate of glucose uptake and catalytic reactions. Among the glucose transporter family, Glut1−4 are responsible for cellular glucose uptake. Unlike other glucose transporters (Glut2−4) with defined tissue localization, Glut1 is the most widely distributed glucose transporter in humans (53 – 57). Moreover, Glut1 and Glut3 possess the highest affinity for glucose and the maximum transport capacity, whereas those of Glut2 are the lowest (58). In this study, we found that *GLUT1* and *GLUT3* mRNA were abundant in HEp-2 cells. In contrast, the mRNA levels of *GLUT2* and *GLUT4* were considerably lower than those of *GLUT1* and *GLUT3* (Fig. 2A through D). Furthermore, RSV infection of HEp-2 cells upregulated Glut1, Glut3, and Glut4 and promoted their transport to the cell membrane, but did not affect Glut2 (Fig. 2E through I; Fig. S3). Therefore, RSV enhanced the ability of HEp-2 cells to take up glucose mainly via Glut1, Glut3, and Glut4, rather than Glut2. Second, the catalytic reactions of glucose in cells are regulated by critical glycolytic enzymes, including HK, PFK, and PK (30 – 32). In this study, we showed that RSV enhanced glycolysis by upregulating the glycolytic enzymes, HK1/2 and PFKP, which are the first and third enzymes in this pathway and are the key rate-limiting enzymes that limit the overall glycolytic rate (Fig. 2J through P) (59, 60). In contrast, RSV did not apparently affect the expression of PKM1/2, PKM2, or LDHA, which are involved in the final steps of glycolysis (Fig. S4) (61, 62). Combined with the promotion of Gluts, RSV infection enhanced glycolysis by promoting glucose uptake and the early stages of glycolysis in infected cells.

HIF-1α acts as a core factor in the regulation of glycolysis and controls the transcription of related Gluts and glycolytic enzymes (13 – 17). Previous studies have reported that HIF-1α-mediated glycolysis is closely associated with infection of host cells by many viruses, such as SARS-CoV-2, WSSV, and ARV (21 – 23). Herein, we confirmed the necessity and importance of HIF-1α in glycolysis during RSV infection. RSV infection contributed to HIF-1α stabilization and upregulated the expression and transcriptional activity of HIF-1α (Fig. 3). Correspondingly, HIF-1α inhibition reversed the RSV-induced increase in Glut1, Glut3, Glut4, HK1, HK2, and PFKP, thereby inhibiting glucose uptake and glycolysis (Fig. 4).

The stability and expression of HIF-1α are regulated by multiple cellular components, functions, and signaling pathways. First, activation of the PI3K/Akt axis in cells is sufficient for the protein synthesis and activation of HIF-1α (19, 20, 36). The PI3K/Akt axis is the main downstream pathway for IR to perform its functions (34, 35). IRS-1 couples signaling from IR to PI3K-dependent pathways. Upon phosphorylation by IR, IRS1 is predicted to bind to the p85α regulatory subunit, which results in the activation of the PI3K signaling pathway (40). mTOR acts as its downstream effector and mediates protein synthesis via phosphorylation of 4E-BP1 (42). This study confirmed that the translation and activity of HIF-1α are strictly regulated by IR/PI3K/Akt signaling during RSV infection. RSV infection dramatically increased the level of IR, promoted the interaction between p85α and IRS1, and upregulated the phosphorylation levels of IRS1, Akt, mTOR, and 4E-BP1, indicating the activation of IR/PI3K/Akt signaling. Inhibition of IR or PI3K activity decreased glucose uptake and glycolysis in infected HEp-2 cells because the translation and transcriptional activity of HIF-1α were downregulated after BMS-754807 or LY294002 treatment (Fig. 5; Fig. S6). Second, ROS has been shown to contribute to the stabilization and activation of HIF-1α (18, 43). Mitochondria are integral to ROS production in eukaryotic cells. Once the mitochondria are damaged under the stimulation of environmental stress or pathogens, the levels of ROS in host cells increase dramatically. Interestingly, we found that the stability and activation of HIF-1α also depended on the ROS content in RSV-infected HEp-2 cells. Elevated ROS levels were closely associated with mitochondrial damage following RSV infection. Consequently, all these factors contributed to shift mitochondrial bioenergetic metabolism toward glycolysis (Fig. 6; Fig. S7). Collectively, HIF-1α-mediated glycolysis is regulated by IR/PI3K/Akt signaling, mitochondrial function, and ROS during RSV infection.

Previous studies have demonstrated that RSV requires energy support in the process of infecting host cells such as the fusion phase (63, 64). Thus, RSV must manipulate host cells in different ways to obtain energy. Blocking the energy supply channel may effectively inhibit RSV infection. As expected, we found that HIF-1α inhibition significantly inhibited RSV infection both *in vitro* and *in vivo* due to the inhibition of glycolysis in cells as well as in mouse lung tissues. Mechanistically, the energy generated by HIF-1α-mediated glycolysis mainly acted on the stage of progeny virion production (probably the assembly/budding stage) without affecting RSV entry, genome transcription, or translation (Fig. 7; Fig. S9). However, whether/how HIF-1α precisely regulates the assembly or budding of progeny RSV virions remains to be further explored. Interestingly, although PX-478 was not as effective as ribavirin in inhibiting RSV replication *in vivo*, it was superior to ribavirin in the treatment of RSV-induced inflammation due to the fact that one of the functions of HIF-1α is to regulate the host immune response (Fig. 8).

**Fig 9:**
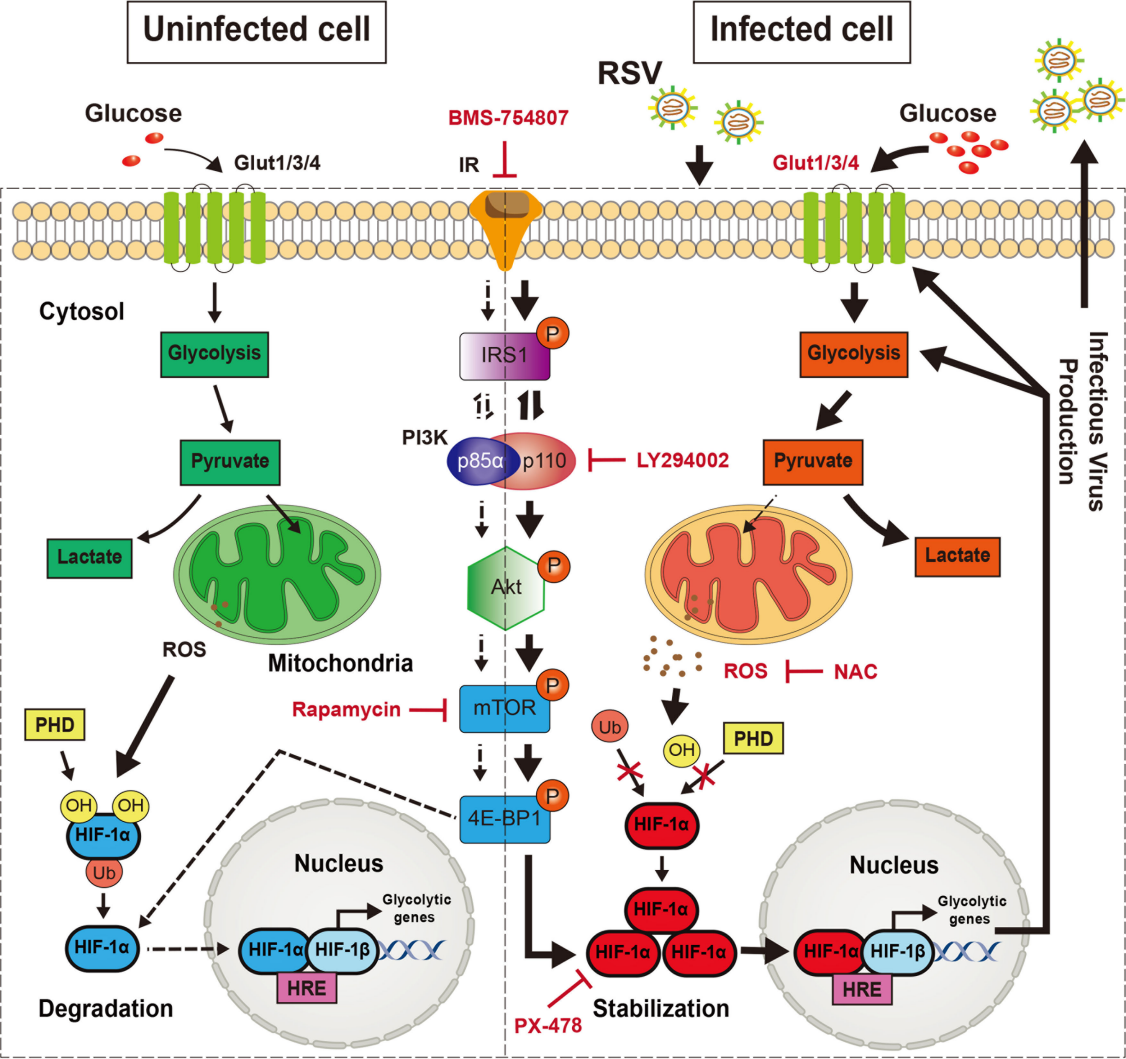
Diagram depicting RSV-driven HIF-1α-mediated glycolysis to facilitates infectious virus production. RSV promotes HIF-1α-mediated glycolysis in infected cells. Upregulation of glycolysis can compensate for the lack of ATP production by damaged mitochondria. In brief, RSV infection increases HIF-1α translation by activating IR/PI3K/Akt signaling. On the other hand, the upregulation of intracellular ROS levels inhibits PHD-mediated HIF-1α hydroxylation, thereby promoting HIF-1α stabilization. Consequently, these events lead to HIF-1α activation and expression of its target genes (GLUTs and glycolytic enzymes), and shift mitochondrial bioenergetic metabolism toward glycolysis, which promote the production of progeny RSV virions. Inhibition of HIF-1α effectively reverses RSV-induced increase in glycolysis, thereby blocking RSV infection.

In summary, RSV infection increases glycolysis *in vitro* and *in vivo*. Among the numerous Gluts and glycolytic enzymes, RSV promotes glucose uptake and glycolysis mainly by upregulating Glut1, Glut3, Glut4, HK1, HK2, and PFKP, independent of Glut2, PKM1, PKM2, and LDHA. Increased glucose uptake and glycolysis induced by RSV depend on the stabilization, expression, and activation of HIF-1α, which is regulated by IR/PI3K/Akt signaling, mitochondrial function, and ROS. Importantly, HIF-1α-mediated glycolysis provides energy for the production of progeny RSV virions. Inhibition of HIF-1α effectively blocks RSV infection both *in vitro* and *in vivo* (Fig. 9). Thus, this study presents a novel and interesting idea for HIF-1α-mediated glycolysis during RSV infection and reveals an effective target for the development of highly efficient anti-RSV drugs.

## MATERIALS AND METHODS

### Cells and virus

HEp-2, Hela, A549, 16HBE, and HEK293T cells were obtained from the American Type Culture Collection (ATCC). RSV A2 (ATCC VR-1540) was acquired from the Medicinal Virology Institute of Wuhan University (Hubei, China). HEp-2, Hela, A549, 16HBE, and HEK293T cells were grown in Dulbecco’s modified Eagle’s medium supplemented with 10% fetal bovine serum and 100 U/mL penicillin-streptomycin. RSV was cultured in HEp-2 cells. The viral titer was measured using a plaque assay, and viral stocks were stored at −80℃.

### Reagents and antibodies

PX-478, BMS-754807, LY294002, fasentin, acetylcysteine (NAC), rapamycin (RAPA), MG-132, and ribavirin were purchased from MedChemExpress (Monmouth Junction, NJ, USA). Anti-glucose transporters (Glut1, Glut2, Glut3, and Glut4), anti-HIF-1α, anti-insulin receptor alpha, anti-insulin receptor beta, anti-p-IRS1 (phospho Y632), anti-IRS1, anti-respiratory syncytial virus fusion (F) glycoprotein, anti-respiratory syncytial virus G glycoprotein, anti-respiratory syncytial virus nucleoprotein, anti-beta actin antibody, goat anti-rabbit IgG H&L (Alexa Fluor 488), and goat anti-mouse IgG H&L (Alexa Fluor 488) were obtained from Abcam (Cambridge, UK). The glycolysis antibody sampler kit, mTOR substrates antibody sampler kit, anti-hydroxy-HIF-1α antibody, anti-p-Akt (Ser473), anti-Akt, anti-mouse IgG horse radish peroxidase (HRP)-linked antibody, and anti-rabbit IgG HRP-linked antibody were acquired from Cell Signaling Technology (Danvers, MA, USA). Anti-PCNA antibody was obtained from Santa Cruz Biotechnology (Dallas, TX, USA). The Dual-Lumi luciferase reporter gene assay kit and reactive oxygen species assay kit were obtained from Beyotime Biotechnology (Shanghai, China). The 2-(N-(7-nitrobenz-2-oxa-1, 3-diazol-4-yl) amino)-2-deoxyglucose (2-NBDG) glucose uptake assay kit was purchased from Abnova (Taiwan, China). The Seahorse XF glycolysis stress test kit and Seahorse XF real-time ATP rate assay kit were acquired from Agilent Technologies Inc. (Santa Clara, CA, USA). The lactate assay kit and glucose oxidation assay kit were acquired from Nanjing Jiancheng Bioengineering Institute (Nanjing, China) and Applygen Technologies Inc. (Beijing, China), respectively.

### Cell treatments

Cells, plasmid-transfected cells, or knocked-down cells were mock-infected or infected with RSV (multiplicity of infection, MOI = 1) in the absence or presence of inhibitors for the indicated durations.

### Plasmids, siRNA, and transfection

EGFP-p85α, mCherry-IRS1, pCMV-HIF1A-3×FLAG Neo (HIF-1α/WT), HRE-luciferase, and pRL-TK plasmids were constructed by MiaoLingBio (Hubei, China). Myc-GLUT4-mCherry was acquired from Addgene (MA, USA). si-HIF-1α was obtained from RiboBio Co. Ltd. (Guangzhou, China). The sequence of the si-HIF-1α is shown in Table S1.

Plasmids or siRNAs were transfected into HEp-2 or HEK293T cells using Lipofectamine 6000 transfection reagent (Beyotime Biotechnology) according to the manufacturer’s instructions. Plasmids or siRNAs were added to Opti-MEM, incubated for 5 min, and then mixed with Opti-MEM containing Lipofectamine 6000. After 20 min of incubation, the mixture was added to cells. At 6-h post-transfection, the mixture was removed and supplemented with culture medium. The related experiments were performed 48 h after transfection.

### Glucose uptake assay

Glucose uptake in cultured cells was measured using a 2-NBDG glucose uptake assay kit according to the manufacturer’s instructions. HEp-2 cells were mock-infected or infected with RSV (MOI = 1) in the absence or presence of inhibitors for 0, 2, 6, 12, or 24 h. The 96-well plate was centrifuged at 1,000 × *g* for 5 min. After removing the supernatant, HEp-2 cells were treated with 2-NBDG staining solution (100 µM) for 20 min. The 2-NBDG staining solution was then aspirated, and the cells were washed once with assay buffer I supplied by the kit. The fluorescence signal was monitored using the FITC channel of a flow cytometer (BD Biosciences, San Jose, CA, USA).

### Measurements of lactic acid production and glucose consumption

Lactic acid and glucose concentrations in the culture supernatant were determined using a lactic acid assay kit and glucose oxidation assay kit, respectively, according to the manufacturer’s instructions. HEp-2 cells were mock-infected or infected with RSV (MOI = 1) in the absence or presence of inhibitors for 0, 2, 6, 12, or 24 h. The supernatant was then transferred to a 96-well plate. In the lactic acid assay, the enzyme working solution and chromogenic reagents were successively added to each well. For the glucose oxidation assay, the working solution was added to each well. After 10 or 20 min of incubation at 37°C, the absorbance values were measured using a microplate reader (Thermo Fisher Scientific, Waltham, MA, USA) at 530 nm.

### Seahorse XF cell glycolysis stress and real-time ATP rate measurements

Glycolytic activity and real-time ATP production rate were measured using an Agilent Seahorse flux analyzer XFe96 according to the manufacturer’s instructions. Briefly, HEp-2 cells were seeded in Seahorse 96-well plates and incubated overnight at 37°C. The cells were mock-infected or infected with RSV (MOI = 1) in the presence or absence of inhibitors for 12 or 24 h. Then, the cells were washed with Seahorse XF assay medium (pH 7.4) containing 2 mM glutamine and incubated in Seahorse XF assay medium for 60 min. In the glycolysis stress test assay, glucose (10 mM), oligomycin (1 µM), and 2-deoxy-D-glucose (2-DG, 50 mM) were injected into ports A, B, and C of the hydrate cartridge, respectively. For the Seahorse XF real-time ATP rate assay, oligomycin (1.5 µM) and Rot/AA (0.5 µM) were injected into ports A and B of the hydrate cartridge, respectively. Finally, the assay was run on an Agilent Seahorse flux analyzer XFe96, and the results were analyzed using Wave Desktop and Report Generator software (Agilent Technologies Inc.).

### Reverse transcription-PCR (RT-PCR)

Total RNA was extracted from the cells using TRIzol reagent (Invitrogen, Carlsbad, CA, USA), and cDNA was obtained using a PrimeScript RT reagent kit (TaKaRa Bio Inc., Kusatsu, Japan) according to the manufacturer’s instructions. Quantitative RT-PCR was performed using a fluorescence-based quantitative PCR system (Roche, Pleasanton, CA, USA) with TB Green Premix Ex Taq (TaKaRa Bio Inc.). The sequences of the primer pairs are shown in Table S2.

### Membrane protein extraction assay

Membrane proteins were extracted using a membrane and cytosol protein extraction kit (Beyotime Biotechnology) according to the manufacturer’s instructions. Briefly, HEp-2 cells were infected with RSV (MOI = 1) for 0, 2, 6, 12, or 24 h. The cells were washed with phosphate-buffered saline (PBS) and collected via centrifugation at 600 × *g* at 4°C for 5 min. The cells were then exposed to membrane protein extraction reagent A containing phenylmethylsulfonyl fluoride (PMSF) and incubated at 4°C for 15 min. The cell suspension was transferred to a homogenizer and homogenized 50 times at 4°C. The cytosolic proteins were collected via centrifugation at 14,000 × *g* at 4°C for 30 min, followed by the removal of nuclei and unbroken cells via centrifugation at 700 × *g* at 4°C for 10 min. The pellet was incubated with membrane protein extraction reagent B at 4°C for 10 min. This step was repeated two times. The membrane proteins were collected via centrifugation at 14,000 × *g* at 4°C for 5 min. The samples were analyzed using western blotting.

### Nuclear and cytosolic protein extraction assay

Nuclear and cytosolic proteins were extracted using a nuclear protein extraction kit (Solarbio, Beijing, China) according to the manufacturer’s instructions. Briefly, HEp-2 cells were infected with RSV (MOI = 1) for 0, 2, 6, 12, or 24 h. Then, the cells were washed with PBS and collected via centrifugation at 500 × *g* at 4°C for 3 min. Next, the cells were exposed to cytosolic protein extraction reagent containing 1 mM PMSF and incubated at 4°C for 10 min. The cell suspension was vortexed for 10 s, and the cytosolic proteins were collected via centrifugation at 15,000 × *g* at 4°C for 10 min. The pellet was incubated in the nuclear protein extraction reagent at 4°C for 10 min. The nuclear proteins were collected via centrifugation at 15,000 × *g* at 4°C for 10 min. The samples were analyzed using western blotting.

### Western blot assay

The cells were lysed in a cell lysis mixture containing cell lysis solution, 20 × protease inhibitor, 20 × protease-phosphatase inhibitor, and 100 × PMSF at 4°C for 30 min. The total proteins were denatured, separated using sodium dodecyl sulfate-polyacrylamide gel electrophoresis, and transferred to polyvinylidene fluoride membranes. The membranes were blocked, probed with primary antibodies overnight at 4°C, and incubated with secondary antibodies for 2 h at room temperature. The protein bands were visualized using enhanced chemiluminescence with an Amersham Imager 600 (General Electric Co., Boston, MA, USA) and quantified using the ImageJ software (National Institutes of Health, Bethesda, MD, USA).

### Immunofluorescence assay

The cell monolayer was fixed with 4% paraformaldehyde for 60 min. The cells were then permeabilized using 0.1% Triton X-100 in PBS for 15 min, followed by incubation with 5% bovine serum albumin for 60 min at 37°C. The cells were then stained with the indicated antibodies overnight at 4°C before being treated with the indicated fluorescent secondary antibodies for 60 min at 37°C. Nuclear DNA was labeled with 4′,6-diamidino-2-phenylindole (DAPI). The cells were washed three times at the end of each process. Finally, the fluorescence intensity was measured, and the images were acquired using a confocal microscope (Carl Zeiss AG, Oberkochen, Germany).

### Dual-luciferase reporter assay

HEK293T cells were transfected with the HRE-luciferase and pRL-TK plasmids using Lipofectamine 6000 as described above. After 48 h of transfection, the cells were mock-infected or infected with RSV (MOI = 1) in the absence or presence of inhibitors for the indicated durations. The activities of firefly and Renilla luciferase were detected using a dual-luciferase reporter assay system (Promega, WI, USA).

### Cytotoxicity assay

The cytotoxicity of si-HIF-1α was determined using the cell counting kit-8 (CCK-8) assay according to the manufacturer’s instructions (MedChemExpress). Briefly, confluent HEp-2 cells in a 96-well plate were transfected with si-NC (50 nM) or si-HIF-1α (50 nM) and incubated at 37°C for 48 h. Next, 10 µL of CCK-8 reagent was added to each well. After 30 min of incubation at 37°C, the absorbance values were measured using a microplate reader (Thermo Fisher Scientific) at 450 nm. Cell viability was expressed as a percentage of non-transfected controls.

### Transmission electron microscopy

The effect of RSV on the mitochondria was determined using TEM. Briefly, HEp-2 cells were seeded in a six-well plate and incubated overnight at 37°C. The cells were mock-infected or infected with RSV (MOI = 1) for 24 h, washed three times with PBS, and fixed with 2.5% glutaraldehyde at 4°C for 30 min. The pellet was dehydrated using an acetone series and embedded in epoxy resin. Images of the mitochondria were acquired using a Hitachi H-7500 (HITACHI, Tokyo, Japan).

### ROS measurement assay

HEp-2 cells were mock-infected or infected with RSV (MOI = 1) for 0, 2, 6, 12, or 24 h. The supernatant was removed and HEp-2 cells were treated with DCFH-DA (10 µM) at 37°C for 20 min. The cells were then washed thrice with serum-free culture medium and the absorbance values were read on a microplate reader (Thermo Fisher Scientific).

### Plaque assay

Confluent HEp-2 cells in 24-well plates were inoculated with the viral supernatant from mouse lung tissues in each group at 37°C for 2 h. The monolayer was then washed once with PBS and overlaid with culture medium containing 1.2% agarose. After 4 days of incubation, the monolayer was fixed in 4% formalin for 2 h. The agarose was removed, and the monolayer was exposed to 1% crystal violet. After 30 min of incubation, the plaques were visualized and recorded.

### Animals and experimental protocol

Three-week-old BALB/c mice (Guangdong Medical Laboratory Animal Center, Guangzhou, China) were randomized into four groups (mock, RSV, RSV + ribavirin, and RSV + PX-478 groups) and intranasally infected with RSV. Ribavirin (40 mg·kg^−1^) and PX-478 (20 mg·kg^−1^) were intragastrically administered to two groups of 10 mice each. The other two groups received equal volumes of normal saline. The compounds were intragastrically administered every 24 h from −1 day before to 3 days following RSV infection. On day 4 after RSV infection, the mice were sacrificed and the lung tissues were excised aseptically. The lung tissues were used for RT-PCR, immunofluorescence staining, plaque assays, and histopathological examination. Blood samples were obtained and used to determine the levels of inflammatory cells. All mice were handled in strict adherence to the Guidelines for Laboratory Animal Use and Care of the Chinese Center for Disease Control and Prevention and the Rules for Medical Laboratory Animals of the Ministry of Health, China. The animal study protocol was approved by the Ethics Committee of Jinan University and the National Institute for Communicable Disease Control and Prevention. The institution reference ID for animal ethics approval is 20220226-15.

### Statistical analysis

Significant differences between groups were determined using Student’s *t* test or one-way analysis of variance (ANOVA) followed by Tukey’s test. Results are presented as the mean ± SD using GraphPad Prism v.9.4 software (GraphPad Software, La Jolla, CA, USA). *P* < 0.05 was considered statistically significant.

## SUPPLEMENTAL MATERIAL

Supplemental material mbio.02110-23-s0001.pdf Supplemental text, Tables S1 and S2, and Fig. S1 to S10.

ASM does not own the copyrights to Supplemental Material that may be linked to, or accessed through, an article. The authors have granted ASM a non-exclusive, world-wide license to publish the Supplemental Material files. Please contact the corresponding author directly for reuse.
