# Supplementary material for: Respiratory syncytial virus co-opts hypoxia-inducible factor-1α-mediated glycolysis to favor the production of infectious virus
Source: mBio. 2023 Oct 5;14(5):e02110-23. doi: 10.1128/mbio.02110-23 (PMC10653832; doi:10.1128/mbio.02110-23)
Supplement: Supplemental material — Supplemental text, Tables S1 and S2, and Fig. S1 to S10. [file mbio.02110-23-s0001.pdf]

Supplementary material

MATERIALS AND METHODS

Plaque assay. Confluent HEp-2 cells in 24-well plates were inoculated with the viral supernatant of each group at 37 °C for 2 h. The monolayer was then washed once with PBS and overlaid with culture medium containing 1.2% agarose. After 4 days of incubation, the monolayer was fixed in 4% formalin for 2 h. The agarose was removed, and the monolayer was exposed to 1% crystal violet. After 30 min of incubation, the plaques were visualized and recorded.

Seahorse XF cell glycolysis stress measurement. Glycolytic activity was measured using an Agilent Seahorse flux analyzer XFe96 according to the manufacturer's instructions. Briefly, 16HBE cells were seeded in Seahorse 96-well plates and incubated overnight at 37 °C. The cells were mock-infected or infected with RSV (MOI = 1) for 24 h. Then, the cells were washed with Seahorse XF assay medium (pH 7.4) containing 2 mM glutamine and incubated in Seahorse XF assay medium for 60 min. Glucose (10 mM), oligomycin (1 μM), and 2-deoxy-D-glucose (2-DG, 50 mM) were injected into ports A, B, and C of the hydrate cartridge, respectively. Finally, the assay was run on an Agilent Seahorse flux analyzer XFe96, and the results were analyzed using Wave Desktop and Report Generator software (Agilent Technologies Inc.).

Immunofluorescence assay. The cell monolayer was fixed with 4% paraformaldehyde for 60 min. The cells were then permeabilized using 0.1% Triton X-100 in PBS for 15 min, followed by incubation with 5% bovine serum albumin for 60

min at 37 °C. The cells were then stained with the indicated antibodies overnight at 4 °C before being treating with the indicated fluorescent secondary antibodies for 60 min at 37 °C. Nuclear DNA was labeled with 4',6-diamidino-2-phenylindole (DAPI). The cells were washed thrice at the end of each process. Finally, the fluorescence intensity was measured, and the images were acquired using a confocal microscope (Carl Zeiss AG, Oberkochen, Germany).

Reverse transcription-polymerase chain reaction (RT-PCR). Total RNA was extracted from the cells using TRIzol reagent (Invitrogen, Carlsbad, CA, USA), and cDNA was obtained using a PrimeScript™ RT reagent kit (TaKaRa Bio Inc., Kusatsu, Japan) according to the manufacturer's instructions. Quantitative RT-PCR was performed using a fluorescence-based quantitative PCR system (Roche, Pleasanton, CA, USA) with TB Green Premix Ex Taq (TaKaRa Bio Inc.). The sequences of the primer pairs are shown in Table S2.

Western blot assay. The samples from cells or mouse lung tissues were lysed in a lysis mixture containing lysis solution, 20 × protease inhibitor, 20 × protease-phosphatase inhibitor, and 100 × PMSF at 4 °C for 30 min. The total proteins were denatured, separated using sodium dodecyl sulfate-polyacrylamide gel electrophoresis, and transferred to polyvinylidene fluoride membranes. The membranes were blocked, probed with primary antibodies overnight at 4 °C, and incubated with secondary antibodies for 2 h at room temperature. The protein bands were visualized using enhanced chemiluminescence with an Amersham Imager 600 (General Electric Co., Boston, MA,

USA) and quantified using the Image J software (National Institutes of Health, Bethesda, MD, USA).

Dual-luciferase reporter assay. HEK293T cells were transfected with the HRE-luciferase and pRL-TK plasmids using Lipofectamine 6000 as described above. After 48 h of transfection, the cells were mock-infected or infected with RSV (MOI = 1) in the absence or presence of rapamycin (RAPA, 200 nM) for 24 h. The activities of firefly and renilla luciferase were detected using a dual-luciferase reporter assay system (Promega, WI, USA).

Statistical analysis. Significant differences between groups were determined using Student's t-test or one-way analysis of variance (ANOVA) followed by Tukey's test. Results are presented as the mean ± SD using GraphPad Prism v.9.4 software (GraphPad Software, La Jolla, CA, USA). $P < 0.05$ was considered statistically significant.

TABLE S1 siRNA target sequence

siRNA	Target sequence（5'-3'）
si-HIF-1α	GGAACATGATGGTTCACTT

TABLE S2 Primer sequences

Genes	Forward primer (5′–3′)	Reverse primer (5′–3′)
HIF1A	GAACGTCGAAAAGAAAAGTCTCG	CCTTATCAAGATGCGAACTCACA
GLUT1	TCTGGCATCAACGCTGTCTTC	CGATACCGGAGCCAATGGT
GLUT2	ATGTAGGAGGATGTCTTTG	GCTCTGTAGTGTTTGTGTG
GLUT3	GCTGGGCATCGTTGTTGGA	GCACTTTGTAGGATAGCAGGAAG
GLUT4	ATCCTTGGACGATTCCTCATTGG	CAGGTGAGTGGGAGCAATCT
HK1	CCAACATTCGTAAGGTCCATTCC	CCTCGGACTCCATGTGAACATT
HK2	GAGCCACCACTCACCCTACT	CCAGGCATTCGGCAATGTG
PFKP	GACCTTCGTTCTGGAGGTGAT	CACGGTTCTCCGAGAGTTTG
PKM	ATAACGCCTACATGGAAAAGTGT	TAAGCCCATCATCCACGTAGA
LDHA	TTGACCTACGTGGCTTGGAAG	GGTAACGGAATCGGGCTGAAT
INSR	CATCCGGGGATCACGACTG	ATCAGGTTGTAGAGGCCGAGT
IRS1	CCCAGGACCCGCATTCAAA	GGCGGTAGATACCAATCAGGT
AKT	AGCGACGTGGCTATTGTGAAG	GCCATCATTCTTGAGGAGGAAGT
RSV NS1	TGTATGTATCACTGCCTTAGCCAAAG	ATGGGCAGCAATTCATTGAGTATG
RSV P	TTTGCTAAGACTCCCCACCGTA	CTTACTACCCAAGGACATAGCCAAC
RSV L	TATCCACGGTCCCACTCTTAGG	AGTATGCAGGCATAGGCCACA
Human-GAPDH	TGATGACCCTTTTGGCTCCC	AAATCCCATCACCATCTTCCAG
Mouse-GAPDH	AGGTCGGTGTGAACGGATTTG	TGTAGACCATGTAGTTGAGGTCA

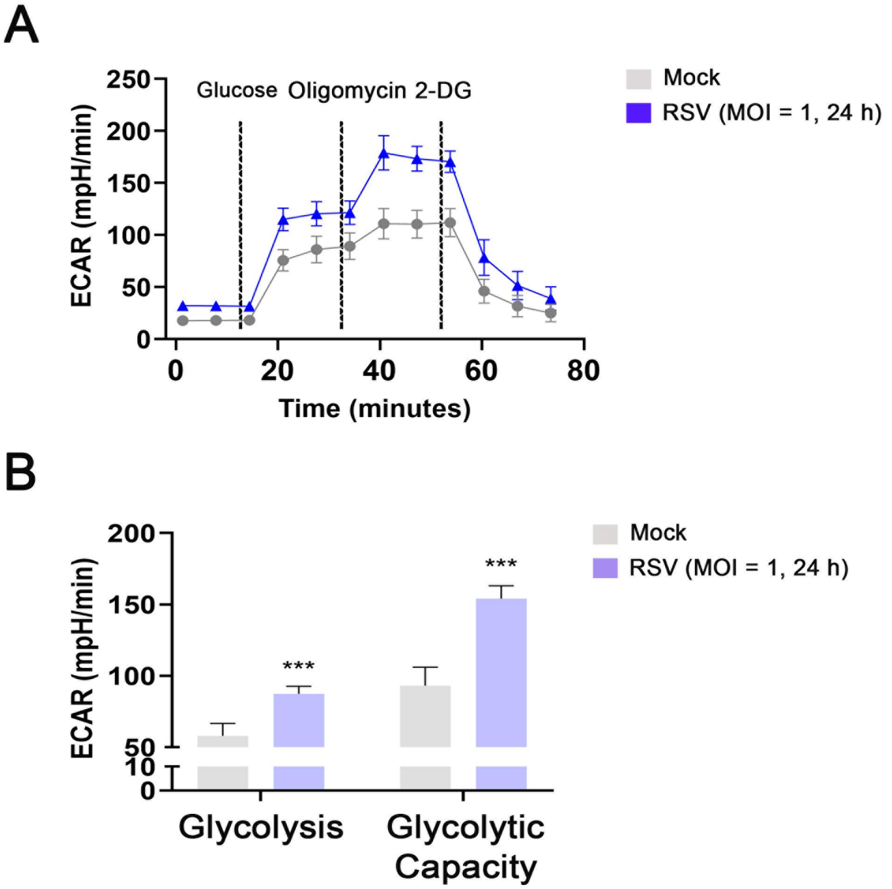

FIG S1 RSV infection elevates glycolysis in infected 16HBE cells. 16HBE cells were mock-infected or infected with RSV (MOI = 1) for 24 h. (A, B) Glycolytic activity was determined using the Seahorse XF glycolysis stress test kit. Data are shown as mean ± SD of at least three biological replicates, statistical analysis using Student's t-test. (***$P <$ 0.001 compared to the blank control group).

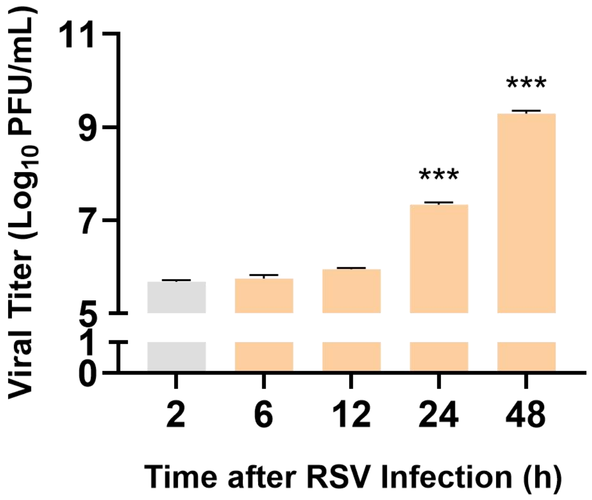

FIG S2 The viral growth kinetics. HEp-2 cells were mock-infected or infected with RSV (MOI = 1) for 2, 6, 12, 24, or 48 h. The viral titer of each group was tested by plaque assay. Data are shown as mean ± SD of three biological replicates, statistical analysis using one-way ANOVA. (****P* < 0.001 compared to the 2 h group).

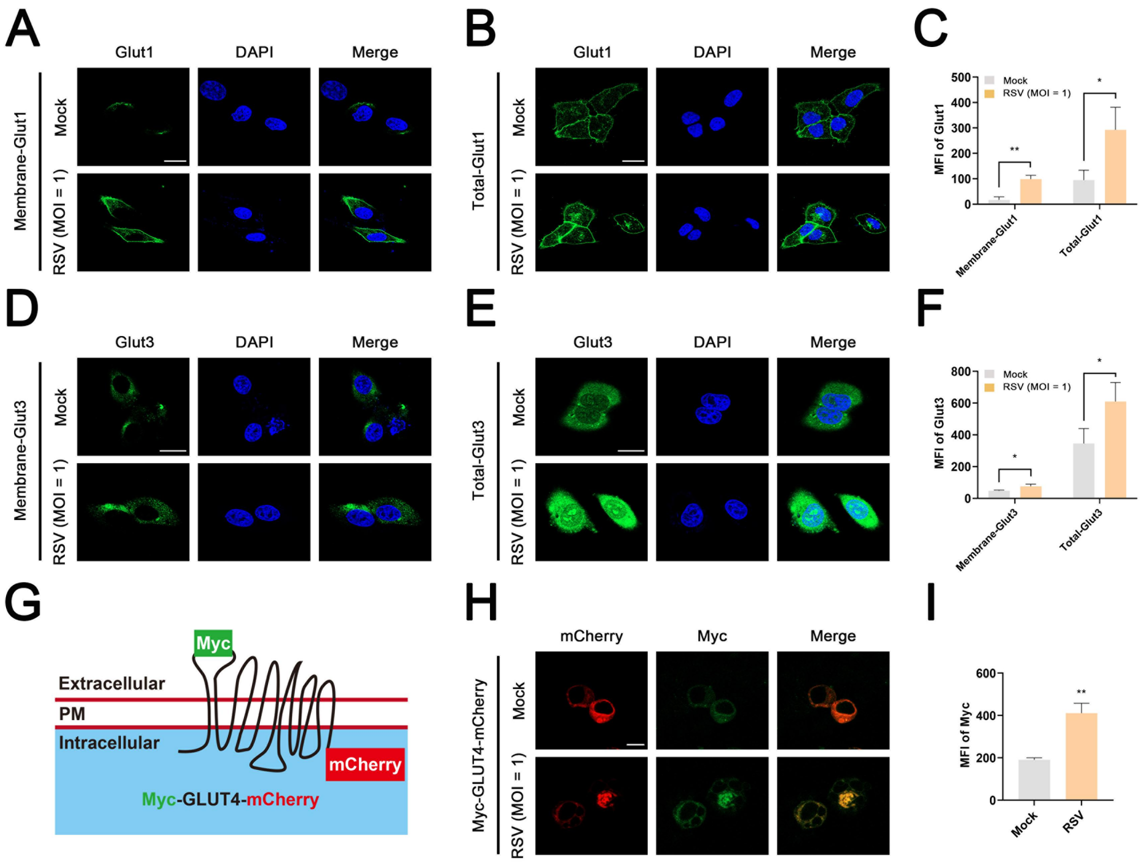

FIG S3 RSV infection increases the expression of Glut1, Glut3, and Glut4 and their translocation to the cell membrane in infected cells. (A-F) HEp-2 cells were mock-infected or infected with RSV (MOI = 1) for 24 h. The fluorescence intensity of Glut1 or Glut3 in the membrane fraction and the whole cell was measured using a confocal microscope. (G-I) HEK293T cells transfected with Myc-GLUT4-mCherry were mock-infected or infected with RSV (MOI = 1) for 24 h. The fluorescence intensity of Myc-GLUT4-mCherry was measured using a confocal microscope. Myc-GLUT4-mCherry: the Myc epitope was inserted in the N terminus of Glut4, and mCherry fused at the C terminus, thus allowing detection of Glut4 in the membrane faction (by Myc) and of total Glut4 content (by mCherry). Scale bar: 20 μm. Data are

shown as mean ± SD of three biological replicates, statistical analysis using Student's t-test. (*$P < 0.05$, **$P < 0.01$ compared to the blank control group).

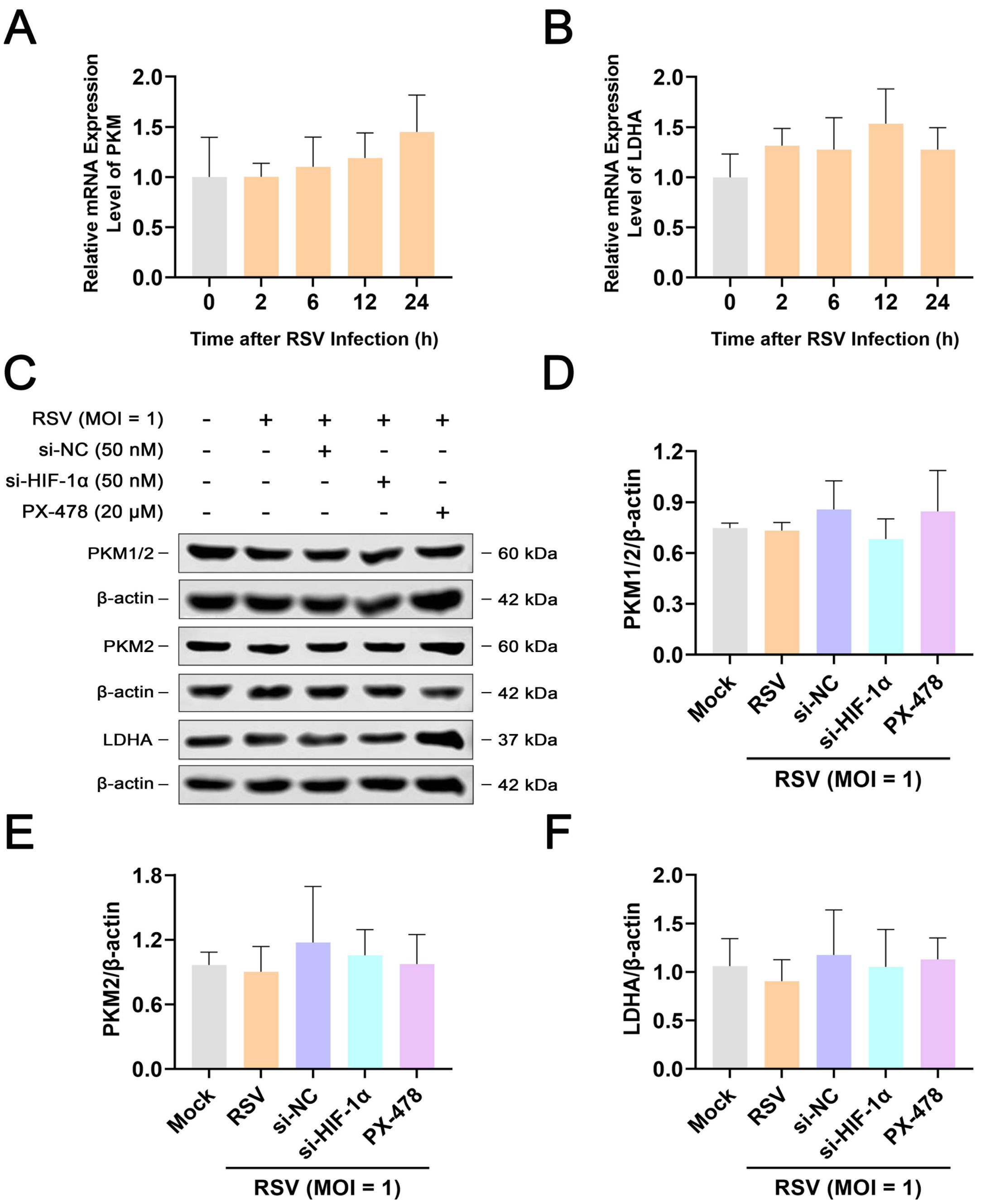

FIG S4 RSV infection has no effect on the expression of PKM and LDHA in infected cells. HEp-2 cells were mock-infected or infected with RSV (MOI = 1) in the presence or absence of si-HIF-1α (50 nM), si-NC (50 nM), or PX-478 (20 μM) for the indicated durations. (A, B) The mRNA levels of *PKM* and *LDHA* 0, 2, 6, 12, or 24 h after RSV

infection were detected using RT-PCR. (C-F) Western blot analysis of PKM1/2, PKM2, and LDHA in the total protein 24 h after RSV infection. Data are shown as mean ± SD of three biological replicates, statistical analysis using one-way ANOVA.

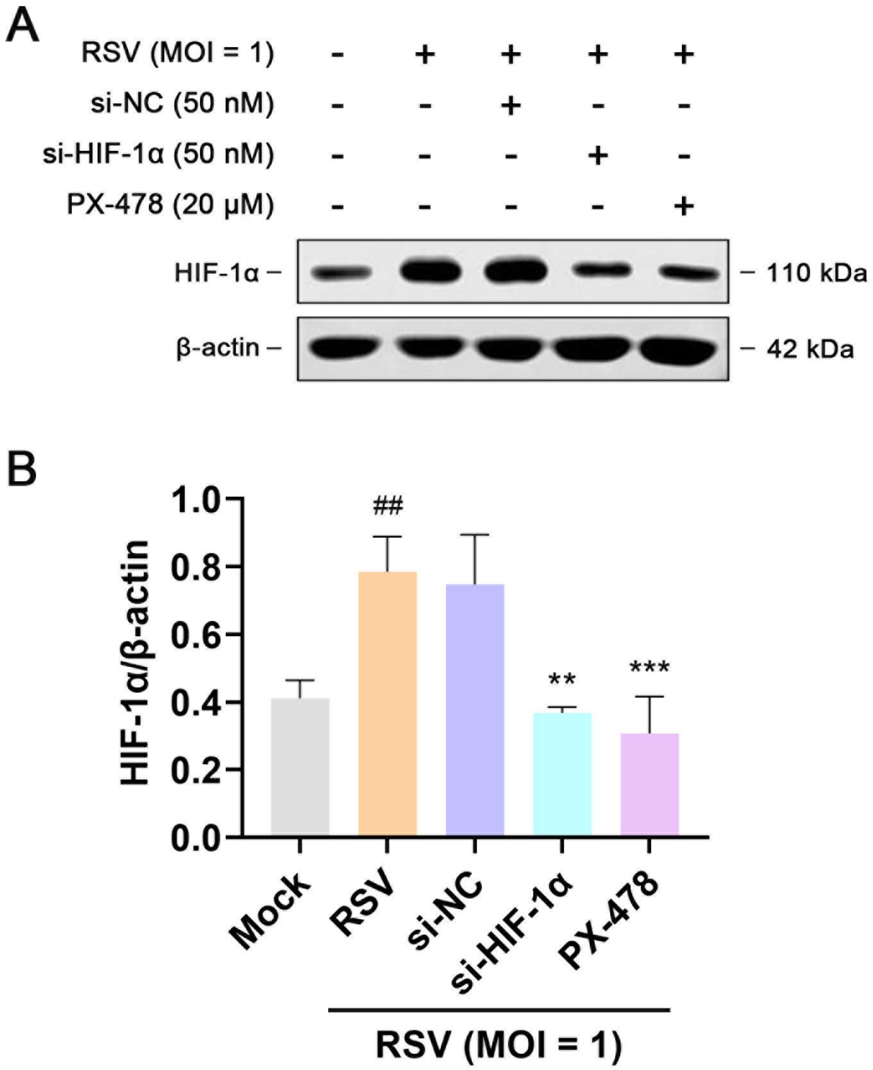

FIG S5 Inhibition of HIF-1α decreases the expression of HIF-1α in infected cells. HEp-2 cells were mock-infected or infected with RSV (MOI = 1) in the presence or absence of si-HIF-1α (50 nM), si-NC (50 nM), or PX-478 (20 μM) for 24 h. The protein level of HIF-1α was determined using western blot assay. Data are shown as mean ± SD of three biological replicates, statistical analysis using one-way ANOVA. ($^{\#\#}P < 0.01$ compared to the blank control group; $^{**}P < 0.01$, $^{***}P < 0.001$ compared to the viral control group).

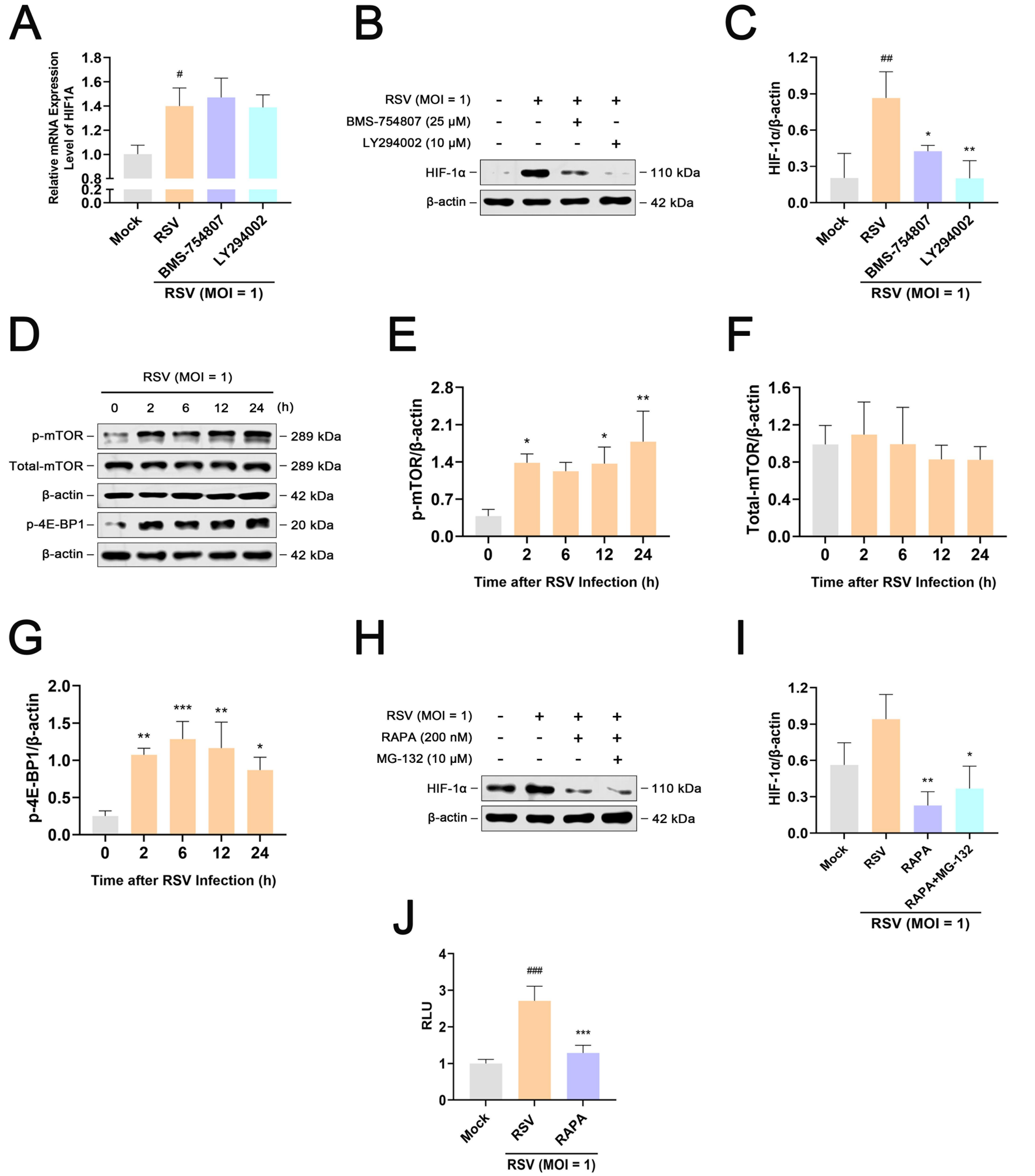

FIG S6 Inhibition of IR/PI3K/Akt signaling decreases HIF-1α translation in infected cells. HEp-2 cells were mock-infected or infected with RSV (MOI = 1) in the presence or absence of BMS-754807 (25 μM), LY294002 (10 μM), rapamycin (RAPA, 200 nM), or MG-132 (10 μM) for the indicated durations. (A) Effect of BMS-754807 or LY294002 on

the mRNA level of *HIF1A* 24 h after RSV infection was detected using RT-PCR. (B, C) Effect of BMS-754807 or LY294002 on the protein expression of HIF-1α 24 h after RSV infection was determined using western blot assay. (D-G) Western blot analysis of p-mTOR, mTOR, and p-4E-BP1 0, 2, 6, 12, or 24 h after RSV infection. (H, I) Effect of RAPA on the protein expression of HIF-1α 24 h after RSV infection was determined using western blot assay. (J) Effect of RAPA on HIF-1α activity 24 h after RSV infection was detected using the dual-luciferase reporter assay system. Data are shown as mean ± SD of at least three biological replicates, statistical analysis using one-way ANOVA. ($^{\#}P < 0.05$, $^{\#\#}P < 0.01$, $^{\#\#\#}P < 0.001$ compared to the blank control group; $^{*}P < 0.05$, $^{**}P < 0.01$, $^{***}P < 0.001$ compared to the 0 h group or the viral control group).

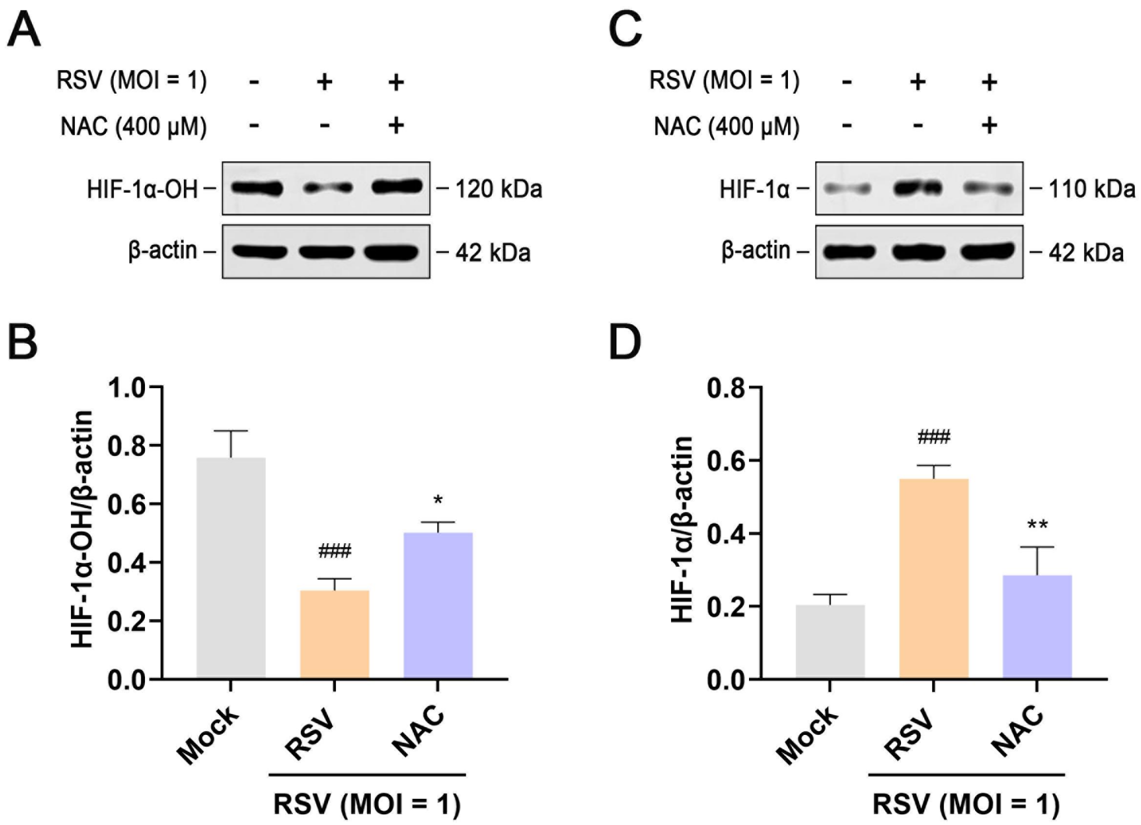

FIG S7 Inhibition of ROS decreases HIF-1α stability in infected cells. HEp-2 cells were mock-infected or infected with RSV (MOI = 1) in the presence or absence of NAC (400 μM) for 24 h. The protein level of HIF-1α-OH or total HIF-1α was determined using western blot assay. Data are shown as mean ± SD of three biological replicates, statistical analysis using one-way ANOVA. ($^{###}P < 0.001$ compared to the blank control group; $^{*}P < 0.05$, $^{**}P < 0.01$ compared to the viral control group).

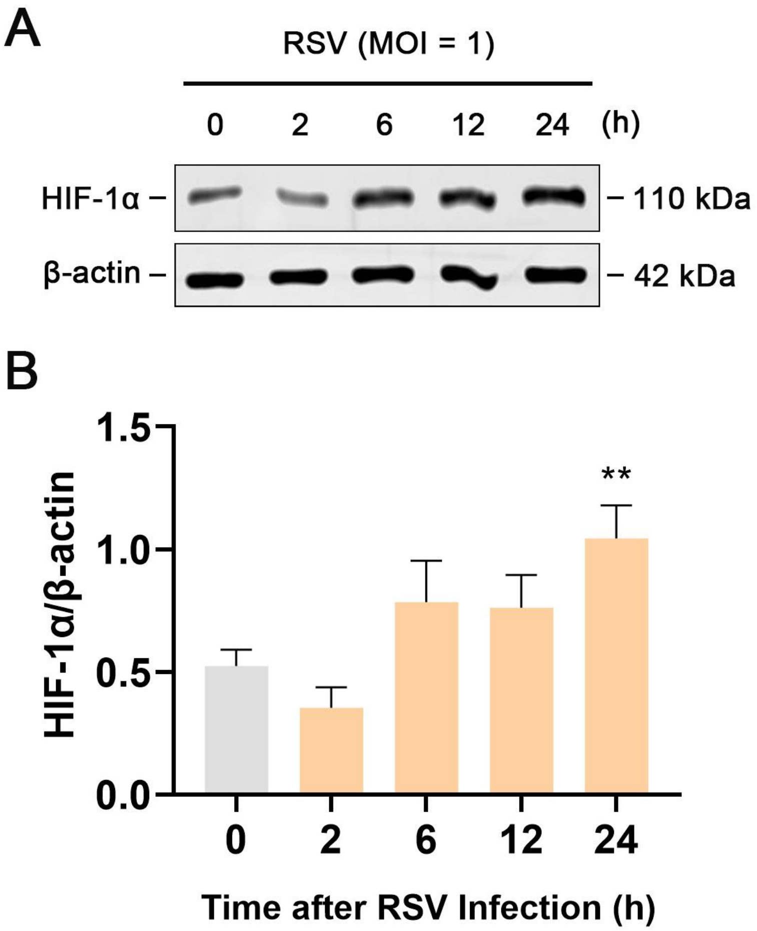

FIG S8 RSV infection increases the expression of HIF-1α in infected A549 cells. (A, B) A549 cells were mock-infected or infected with RSV (MOI = 1) for 0, 2, 6, 12, or 24 h. The protein level of HIF-1α was detected using western blot assay. Data are shown as mean ± SD of three biological replicates, statistical analysis using one-way ANOVA. (***P* < 0.01 compared to the 0 h group).

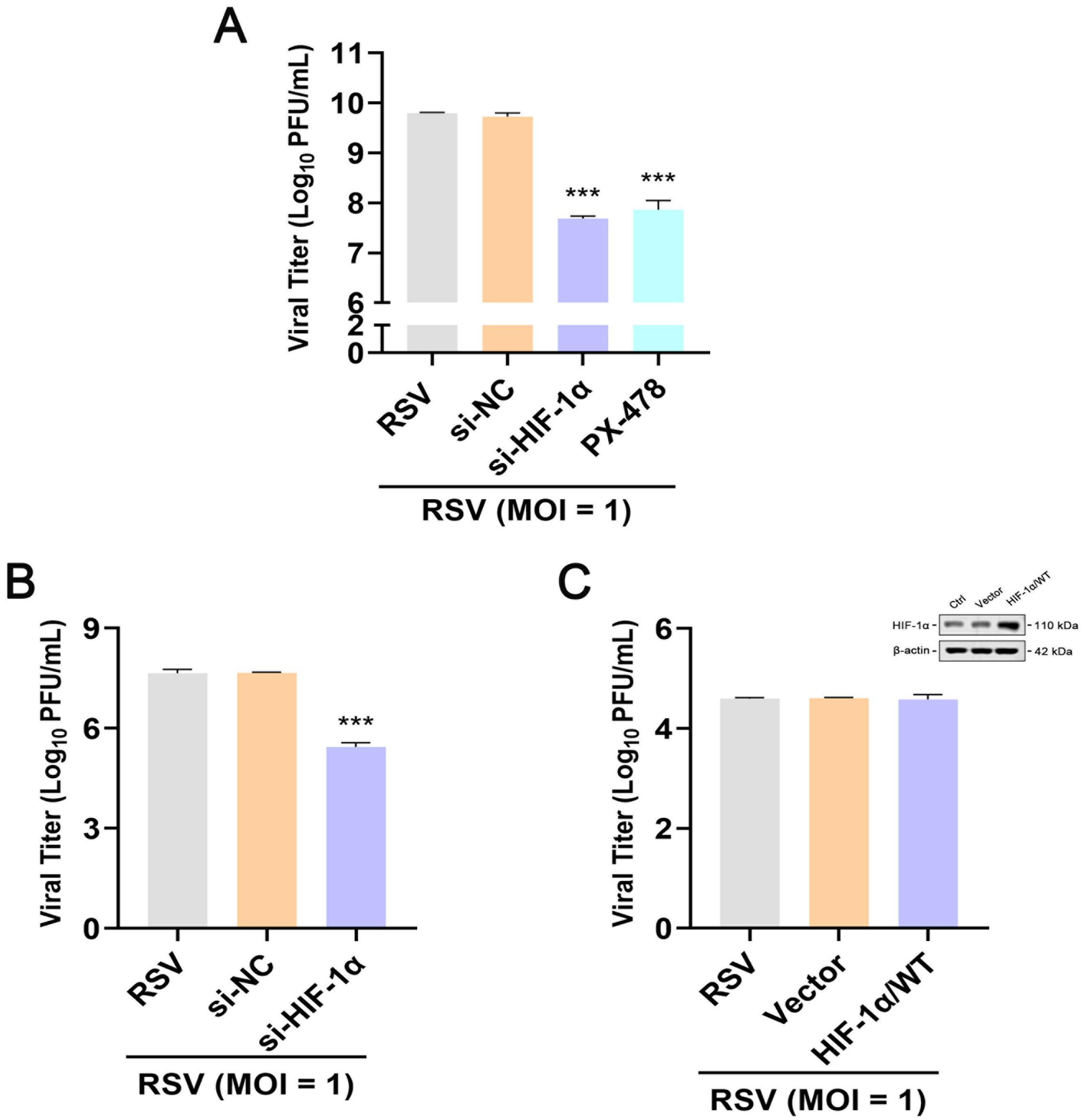

FIG S9 Inhibition of HIF-1α inhibits infectious virus production *in vitro*. (A) HEp-2 cells were mock-infected or infected with RSV (MOI = 1) in the presence or absence of si-HIF-1α (50 nM), si-NC (50 nM), or PX-478 (20 μM) for 48 h. Effect of si-HIF-1α or PX-478 on viral titers in RSV-infected HEp-2 cells was measured using plaque assay. (B) HEp-2 cells transfected with si-HIF-1α (50 nM) or si-NC (50 nM) were mock-infected or infected with RSV (MOI = 1) for 48 h. The supernatant of each group was collected, and

the viral titer was tested by plaque assay. (C) HEK293T cells transfected with HIF-1α or vector plasmid were mock-infected or infected with RSV (MOI = 1) for 48 h. The supernatant of each group was collected, and the viral titer was tested by plaque assay. Data are shown as mean ± SD of three biological replicates, statistical analysis using one-way ANOVA. (***$P < 0.001$ compared to the viral control group).

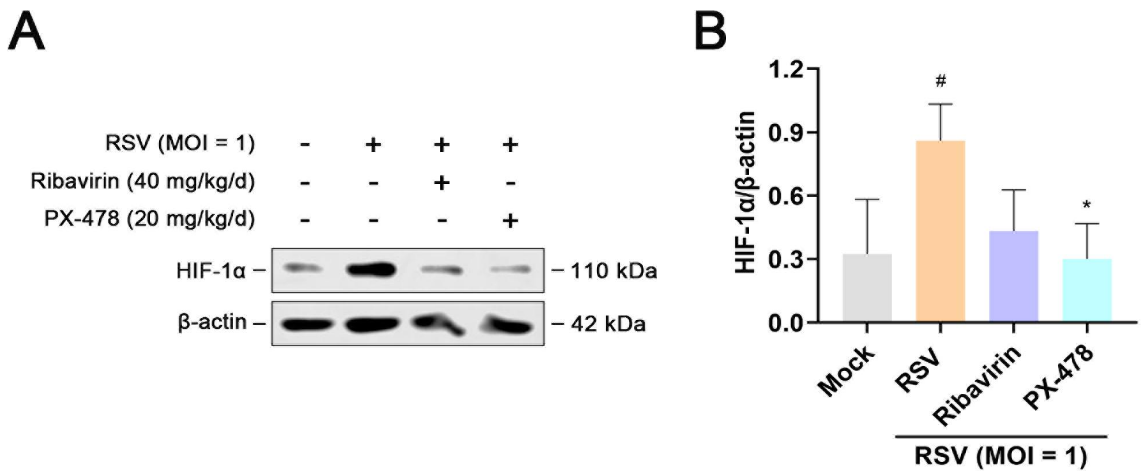

FIG S10 PX-478 inhibits the expression of HIF-1α in the lung tissues. BALB/c mice were mock-infected or infected with RSV. PX-478 (20 mg·kg^{-1}) was intragastrically administered before inoculation of RSV (-1 day) and then administered every 24 h (-1 to 3 days). On day 4 after RSV infection, the protein level of HIF-1α in the lung tissues was detected using western blot assay. Data are shown as mean ± SD of three biological replicates, statistical analysis using one-way ANOVA. ($^{\#}P < 0.05$ compared to the blank control group; $^{*}P < 0.05$ compared to the viral control group).
